# Supplementary material for: Utilizing Heteroatom Types and Numbers from Extensive Ligand Libraries to Develop Novel hERG Blocker QSAR Models Using Machine Learning-Based Classifiers
Source: ACS Omega. 2023 Oct 16;8(43):40864–77. doi: 10.1021/acsomega.3c06074 (PMC10620895; doi:10.1021/acsomega.3c06074)
Supplement: Supplementary file 1 — ao3c06074_si_001.pdf [file ao3c06074_si_001.pdf]

Supporting Information

Utilizing Heteroatom Types and Numbers from Extensive Ligand Libraries to Develop Novel hERG Blocker QSAR Models Using Machine Learning-based Classifiers

Safa Haddad[1,2], Lalehan Oktay[1,2], Ismail Erol[1,2], Kader Şahin[3], Serdar Durdagi[1,2,4,*]

[1]Computational Biology and Molecular Simulations Laboratory, Department of Biophysics, School of Medicine, Bahçeşehir University, Istanbul, Turkey; [2]Computational Drug Design Center (HITMER), Bahçeşehir University, Istanbul, Turkey; [3]Department of Analytical Chemistry, School of Pharmacy, Bahçeşehir University, Istanbul, Turkey; [4]Molecular Therapy Lab, Department

Supporting Tables

Table S1. Results of the top Auto-QSAR traditional method models.

Numeric model	kpls_radial_19	kpls_radial_49	kpls_radial_45
**Ranking score**	0.41	0.50	0.48
**Training set / Test set ratio**	75%-25%	85%-15%	85%-15%
**R^2**	0.79	0.78	0.78
**Q^2**	0.54	0.60	0.57

Table S2. Results of the top Auto-QSAR Deepchem models.

qsar_build	Model code	Training set / Test set ratio	Score	R^2	Q^2
**qsar_build_15**	dl_1	80%-20%	0.28	0.96	0.51
**qsar_build_35**	dl_1	75%-25%	0.33	0.97	0.56
**qsar_build_34**	dl_1	75%-25%	0.37	0.94	0.58

Table S3. Results of the MLR and PLS method models from CANVAS.

approach	Q^2	R^2	Training set/Test set ratio
**MLR**	-0.01	0.08	80%-20%
	0.03	0.06	85%-15%
	0.03	0.07	75%-25%
	0.008	0.08	70%-30%
**PLS**	1.15	0.24	80%-20%
	0.24	0.20	85%-15%
	0.21	0.19	75%-25%
	0.21	0.21	70%-30%

Table S4. Investigation of the influence of Chlorine on the pIC_{50} activity.

Name	Molecular formula	pIC50	Cl	Hetatoms	Median Value
**CHEMBL75880**	C19H35N	5.11	0	1	5.315
**CHEMBL629**	C20H23N	5.52	0	1	
**CHEMBL284348**	C5H6N2	2.36	0	2	5.11
**CHEMBL3**	C10H14N2	3.61	0	2	
**CHEMBL1086033**	C17H22N2	3.67	0	2	
**CHEMBL87045**	C18H18N2	5.06	0	2	
**CHEMBL459176**	C20H22N2	5.16	0	2	
**CHEMBL11**	C19H24N2	5.47	0	2	
**CHEMBL83**	C26H29NO	6.01	0	2	
**CHEMBL1671894**	C27H26N2	7.75	0	2	
**CHEMBL1087**	C15H22N2O	3.81	0	3	5.825
**CHEMBL1083707**	C20H24N2O	4.67	0	3	
**CHEMBL513258**	C19H19FN2	5.05	0	3	
**CHEMBL2325209**	$C26H28NO2^{+}$	5.54	0	3	
**CHEMBL497171**	C15H17N3	5.8	0	3	
**CHEMBL457930**	C20H19N3	5.85	0	3	
**CHEMBL2324248**	$C20H20NO2^{+}$	6.12	0	3	
**CHEMBL1090528**	C19H24N2S	6.21	0	3	
**CHEMBL299499**	C17H22IN3	6.31	0	3	
**CHEMBL607**	C15H21NO2	6.49	0	3	
**CHEMBL70**	C17H19NO3	3	0	4	5.01
**CHEMBL1671896**	C11H14F3N	3.27	0	4	
**CHEMBL640**	C13H21N3O	3.86	0	4	
**CHEMBL219803**	C17H25N3O	3.94	0	4	
**CHEMBL16**	C15H12N2O2	4	0	4	
**CHEMBL3356251**	C24H36N2O2	4.01	0	4	
**CHEMBL219128**	C17H33N3O	4.17	0	4	
**CHEMBL3786542**	C26H26N2O2	4.21	0	4	

Table S4 (cont.d)

**CHEMBL3356249**	**C25H38N2O2**	**4.3**	**0**	**4**	
**CHEMBL219074**	C20H31N3O	4.42	0	4	
**CHEMBL3314426**	C15H14F2N2	4.66	0	4	
**CHEMBL1086273**	C19H23FN2O	4.68	0	4	
**CHEMBL1089159**	C22H23N3O	4.7	0	4	
**CHEMBL2333646**	C19H23BrN2O	4.89	0	4	
**CHEMBL3220615**	C19H21NO3	4.89	0	4	
**CHEMBL3185736**	C22H20N2O2	4.91	0	4	
**CHEMBL517**	C21H29N3O	4.92	0	4	
**CHEMBL4162513**	C35H34N2O2	5.1	0	4	
**CHEMBL562948**	C22H33N3O	5.4	0	4	
**CHEMBL1090175**	C20H25N3O	5.47	0	4	
**CHEMBL1935443**	C20H23N3O	5.68	0	4	
**CHEMBL1093058**	C17H21N3S	5.7	0	4	
**CHEMBL4450665**	C23H28N2O2	5.92	0	4	
**CHEMBL3323181**	C22H24N2OS	6	0	4	
**CHEMBL460893**	C23H25N3O	6.13	0	4	
**CHEMBL1092377**	C19H21FN2S	6.48	0	4	
**CHEMBL1346**	C28H30N2O2	7.1	0	4	
**CHEMBL2046892**	C22H22F2N2	7.4	0	4	
**CHEMBL96153**	C24H31NO3	7.52	0	4	
**CHEMBL1223951**	C21H24N2O2	9.21	0	4	
**CHEMBL1257938**	C22H26N2O2	9.21	0	4	
**CHEMBL1257937**	C22H26N2O2	**9.28**	**0**	**4**	
**CHEMBL1257820**	C23H28N2O2	9.6	0	4	
**CHEMBL1257821**	C22H26N2O2	9.85	0	4	
**CHEMBL1097**	C15H23N3O2	4	0	5	5.22
**CHEMBL4640366**	C33H34N2O3	4.02	0	5	
**CHEMBL4209354**	C15H20N2O2S	4.07	0	5	
**CHEMBL1091879**	C19H25N3O2	4.12	0	5	

Table S4 (cont.d)

**CHEMBL561138**	**C23H27N3O2**	**4.14**	**0**	**5**	
**CHEMBL1258006**	C22H23N3O2	4.27	0	5	
**CHEMBL3787069**	C26H26N2O3	4.35	0	5	
**CHEMBL1094041**	C29H29N3O2	4.55	0	5	
**CHEMBL4213196**	C25H34N4O	4.62	0	5	
**CHEMBL914**	C32H39NO4	4.67	0	5	
**CHEMBL217133**	C18H22N4O	4.68	0	5	
**CHEMBL429115**	C20H24N4O	4.71	0	5	
**CHEMBL2441636**	C22H33N3O2	4.72	0	5	
**CHEMBL1125**	C16H18N4S	4.90	0	5	
**CHEMBL4096683**	C20H22N4O	4.94	0	5	
**CHEMBL237191**	C25H31N5	4.98	0	5	
**CHEMBL565829**	C15H14N4O	5.01	0	5	
**CHEMBL2177304**	C27H24FN3O	5.07	0	5	
**CHEMBL487063**	C23H27N3O2	5.10	0	5	
**CHEMBL378547**	C21H21F2NO2	5.11	0	5	
**CHEMBL485242**	C25H29N3O2	5.14	0	5	
**CHEMBL478615**	C23H27N3O2	5.17	0	5	
**CHEMBL2326478**	C18H18FN3S	5.17	0	5	
**CHEMBL476579**	C24H29N3O2	5.18	0	5	
**CHEMBL479242**	C23H27N3O2	5.18	0	5	
**CHEMBL3121096**	C20H25N3O2	5.22	0	5	
**CHEMBL498572**	C19H22N2O3	5.23	0	5	
**CHEMBL1819137**	C22H25N3O2	5.23	0	5	
**CHEMBL488249**	C24H27N3O2	5.25	0	5	
**CHEMBL4100591**	C27H34F2N2O	**5.29**	**0**	**5**	
**CHEMBL1956112**	C21H28N4S	5.33	0	5	
**CHEMBL487064**	C23H27N3O2	5.55	0	5	
**CHEMBL1110**	C17H18N4O	5.64	0	5	
**CHEMBL478616**	C23H27N3O2	5.8	0	5	

Table S4 (cont.d)

CHEMBL1224697	C22H28N2O2S	5.9	0	5	
**CHEMBL196983**	C21H27NO4	6.1	0	5	
**CHEMBL58387**	C31H38N4O	6.24	0	5	
**CHEMBL3775729**	C26H28N4O	6.28	0	5	
**CHEMBL376488**	C32H31BrN2O2	6.43	0	5	
**CHEMBL1671893**	C23H30N2O3	6.6	0	5	
**CHEMBL1087493**	C17H26N4O	6.63	0	5	
**CHEMBL715**	C17H20N4S	6.74	0	5	
**CHEMBL2324243**	C26H32NO4^{+}	6.8	0	5	
**CHEMBL572163**	C26H31NO4	7.42	0	5	
**CHEMBL61301**	C19H21FN4	7.56	0	5	
**CHEMBL1642487**	C28H28N2O3	9.09	0	5	
**CHEMBL1642479**	C28H28N2O3	9.17	0	5	
**CHEMBL259732**	C20H27N3O2	9.19	0	5	
**CHEMBL1257577**	C19H22N2O2S	9.37	0	5	
**CHEMBL410832**	C23H25N3O2	9.39	0	5	
**CHEMBL1257578**	C20H24N2O2S	9.59	0	5	
**CHEMBL270190**	C25H32N2O4	3.10	0	6	5.92
**CHEMBL519266**	C22H28N2O4	3.11	0	6	
**CHEMBL4212656**	C24H31N5O	4.64	0	6	
**CHEMBL398612**	C26H35FN4O	4.88	0	6	
**CHEMBL460491**	C21H34N4S2	4.88	0	6	
**CHEMBL1823042**	C20H28FN3O2	5	0	6	
**CHEMBL4067770**	C20H21FN4O	5.11	0	6	
**CHEMBL4100776**	C20H22N4OS	5.23	0	6	
**CHEMBL225036**	C24H26N4OS	5.29	0	6	
**CHEMBL1938433**	C23H24N2O4	5.42	0	6	
**CHEMBL4089699**	C19H19N3O2S	5.42	0	6	
**CHEMBL2177904**	C23H16FN5	5.51	0	6	
**CHEMBL1823047**	C22H30N4O2	5.54	0	6	

Table S4 (cont.d)

CHEMBL4065208	C23H22N4O2	5.56	0	6	
**CHEMBL4104102**	C20H22N4OS	5.61	0	6	
**CHEMBL2177905**	C24H16F2N4	5.8	0	6	
**CHEMBL3219616**	C24H30N2O3S	5.8	0	6	
**CHEMBL1224699**	C21H26N2O3S	5.9	0	6	
**CHEMBL561279**	C25H34N2O3S	5.93	0	6	
**CHEMBL2147024**	C22H18N6	5.96	0	6	
**CHEMBL4081790**	C25H19N5O	6.3	0	6	
**CHEMBL556100**	C22H25NO4S	6.33	0	6	
**CHEMBL723**	C24H26N2O4	6.46	0	6	
**CHEMBL4096145**	C22H27N5O	6.52	0	6	
**CHEMBL3774817**	C25H25FN4O	6.59	0	6	
**CHEMBL522456**	CHEMBL522456	7	0	6	
**CHEMBL4173253**	C26H34N2O3S	7.1	0	6	
**CHEMBL3775282**	C26H28N4O2	7.16	0	6	
**CHEMBL1108**	C22H22FN3O2	7.49	0	6	
**CHEMBL329067**	C32H33N5O	7.62	0	6	
**CHEMBL533**	C20H36N2O3S	8	0	6	
**CHEMBL12780**	C24H27FN4O	8.16	0	6	
**CHEMBL1423**	C28H29F2N3O	8.52	0	6	
**CHEMBL296419**	C28H31FN4O	9.05	0	6	
**CHEMBL1258503**	C22H25N3O3	9.14	0	6	
**CHEMBL1258280**	C22H24N2O4	9.21	0	6	
**CHEMBL374731**	C10H14N2O5	2.3	0	7	5.13
**CHEMBL8**	C17H18FN3O3	3.02	0	7	
**CHEMBL4855895**	C21H24FN3O3	3.53	0	7	
**CHEMBL22**	C14H18N4O3	3.62	0	7	
**CHEMBL578834**	C21H25N5OS	3.72	0	7	
**CHEMBL3582294**	C25H18F2N4O	4.03	0	7	
**CHEMBL4224807**	C14H16N6O	4.03	0	7	

Table S4 (cont.d)

CHEMBL388978	C28H26N4O3	4.04	0	7	
**CHEMBL4847738**	C20H23N3O4	4.05	0	7	
**CHEMBL3329814**	C21H28N2O5	4.09	0	7	
**CHEMBL474484**	C13H21N7	4.28	0	7	
**CHEMBL583**	C19H22FN3O3	4.3	0	7	
**CHEMBL218836**	C18H24F3N3O	4.4	0	7	
**CHEMBL2069925**	C21H21N5OS	4.52	0	7	
**CHEMBL1957012**	C26H29N5O2	4.6	0	7	
**CHEMBL575241**	C21H26N6S	4.71	0	7	
**CHEMBL2333636**	C24H28F4N2O	4.75	0	7	
**CHEMBL2180842**	C17H22FN5O	4.8	0	7	
**CHEMBL2314058**	C25H34F3N3O	4.83	0	7	
**CHEMBL3973288**	C20H22FN3O2S	4.84	0	7	
**CHEMBL523374**	C22H23N5O2	4.89	0	7	
**CHEMBL3115194**	C26H32BrN3O3	4.9	0	7	
**CHEMBL3218884**	C28H33N5O2	4.96	0	7	
**CHEMBL2333607**	C25H30F4N2O	5.05	0	7	
**CHEMBL2333615**	C23H26F4N2O	5.05	0	7	
**CHEMBL498042**	C24H23N5O2	5.08	0	7	
**CHEMBL3703271**	C22H23N3O4	5.08	0	7	
**CHEMBL3262625**	C23H26N6O	5.09	0	7	
**CHEMBL246815**	C22H26FN3O3	5.13	0	7	
**CHEMBL523821**	C21H15F2N5	5.16	0	7	
**CHEMBL398478**	C25H28FN3O3	5.21	0	7	
**CHEMBL2178522**	C23H19FN6	5.28	0	7	
**CHEMBL560386**	C23H29NO5S	5.31	0	7	
**CHEMBL497048**	C27H23N5O2	5.36	0	7	
**CHEMBL2147022**	C20H17N7	5.37	0	7	
**CHEMBL2336331**	C24H29N5O2	5.4	0	7	
**CHEMBL1813015**	C20H20N6O	5.42	0	7	

Table S4 (cont.d)

CHEMBL1939739	C32H35FN4O2	5.44	0	7	
**CHEMBL2147021**	C20H15FN6	5.57	0	7	
**CHEMBL246634**	C21H23FN2O4	5.58	0	7	
**CHEMBL2151218**	C25H22FN5O	5.7	0	7	
**CHEMBL3764774**	C28H28N4O3	5.7	0	7	
**CHEMBL243901**	C23H31N5OS	5.9	0	7	
**CHEMBL17423**	C22H25N3O4	5.96	0	7	
**CHEMBL3775387**	C25H27N5O2	5.99	0	7	
**CHEMBL2387265**	C31H29FN2O4	6.09	0	7	
**CHEMBL1079578**	C28H31N5OS	6.2	0	7	
**CHEMBL3775050**	C25H24F2N4O	6.2	0	7	
**CHEMBL45816**	C29H38FN3O3	6.24	0	7	
**CHEMBL501480**	C29H25N5O2	6.5	0	7	
**CHEMBL1084400**	C20H21F3N4	6.8	0	7	
**CHEMBL584766**	C23H24FN5S	7	0	7	
**CHEMBL526466**	C25H23N5O2	7.1	0	7	
**CHEMBL2146854**	C23H26N4O3	7.33	0	7	
**CHEMBL384487**	C24H23FN2O4	7.69	0	7	
**CHEMBL4279819**	C16H18N6O	8.07	0	7	
**CHEMBL217593**	C23H23FN2O4	8.27	0	7	
**CHEMBL33**	C18H20FN3O4	3.04	0	8	5.03
**CHEMBL399726**	C20H24N6O2	3.78	0	8	
**CHEMBL31**	C19H22FN3O4	3.89	0	8	
**CHEMBL3804950**	C20H21F2N5O	4.01	0	8	
**CHEMBL4858551**	C19H19FN6O	4.01	0	8	
**CHEMBL520463**	C28H24N4O4	4.05	0	8	
**CHEMBL485123**	C20H23F3N4O	4.07	0	8	
**CHEMBL32**	C21H24FN3O4	4.1	0	8	
**CHEMBL2333619**	C22H27F4N3O	4.1	0	8	
**CHEMBL2180070**	C19H23N7O	4.27	0	8	

Table S4 (cont.d)

CHEMBL193	C17H18N2O6	4.3	0	8	
**CHEMBL256653**	C23H26N6OS	4.3	0	8	
**CHEMBL2382343**	C29H32F2N2O4	4.32	0	8	
**CHEMBL2314064**	C24H32F3N3O2	4.41	0	8	
**CHEMBL3577935**	C23H34F3N3O2	4.5	0	8	
**CHEMBL3787345**	C25H28N4O4	4.51	0	8	
**CHEMBL2164565**	C19H18F3N3O2	4.63	0	8	
**CHEMBL602875**	C26H34N4O4	4.72	0	8	
**CHEMBL1939742**	C30H37N5O2S	4.75	0	8	
**CHEMBL3612814**	C24H28N4O4	4.85	0	8	
**CHEMBL3593771**	C19H19N7O	4.87	0	8	
**CHEMBL257901**	C28H30N6OS	4.9	0	8	
**CHEMBL270239**	C30H34N6OS	4.9	0	8	
**CHEMBL515001**	C21H25FN6O	4.96	0	8	
**CHEMBL1084617**	C24H28N4O4	4.96	0	8	
**CHEMBL2440407**	C23H26N2O5S	5.02	0	8	
**CHEMBL465417**	C25H23N5O3	5.04	0	8	
**CHEMBL3786346**	C25H26N4O4	5.05	0	8	
**CHEMBL3287218**	C29H32N6OS	5.16	0	8	
**CHEMBL262341**	C33H29N7O	5.25	0	8	
**CHEMBL207220**	C24H40N4O3S	5.4	0	8	
**CHEMBL270852**	C32H38N6OS	5.4	0	8	
**CHEMBL271909**	C28H30N6S2	5.5	0	8	
**CHEMBL2181489**	C23H20F3N3O2	5.52	0	8	
**CHEMBL272086**	C32H38N6OS	5.6	0	8	
**CHEMBL2207738**	C23H26N4O3S	5.6	0	8	
**CHEMBL399525**	C19H23N7O	5.75	0	8	
**CHEMBL2178521**	C24H21FN6O	5.75	0	8	
**CHEMBL3415593**	C19H26N6O2	5.77	0	8	
**CHEMBL1080489**	C22H27N5O2S	5.8	0	8	

Table S4 (cont.d)

**CHEMBL478462**	C25H38N6O2	5.84	0	8	
**CHEMBL2441431**	C22H23N3O5	5.89	0	8	
**CHEMBL1621**	C23H27FN4O3	6	0	8	
**CHEMBL515025**	C24H36N6O2	6.15	0	8	
**CHEMBL478011**	C25H38N6O2	6.38	0	8	
**CHEMBL408169**	C28H32N6OS	6.4	0	8	
**CHEMBL94454**	C24H25FN6O	6.46	0	8	
**CHEMBL453894**	C25H26F3N3O2	6.46	0	8	
**CHEMBL244280**	C26H28FN5OS	6.6	0	8	
**CHEMBL256442**	C29H32N6S2	6.8	0	8	
**CHEMBL568571**	C27H26F3N3O2	6.85	0	8	
**CHEMBL397429**	C28H30N6OS	7.3	0	8	
**CHEMBL2146853**	C20H23N5O3	7.6	0	8	
**CHEMBL214021**	C23H21FN2O5	7.82	0	8	
**CHEMBL2324519**	C20H21FN6O2	3.67	0	9	5.11
**CHEMBL3799831**	C25H26FN3O5	4.03	0	9	
**CHEMBL3290351**	C24H27FN6O2	4.05	0	9	
**CHEMBL216323**	C23H19FN2O6	4.07	0	9	
**CHEMBL4092041**	C29H31F3N2O4	4.07	0	9	
**CHEMBL3290344**	C24H26N6O3	4.13	0	9	
**CHEMBL248296**	C27H31F2N3O4	4.19	0	9	
**CHEMBL2165068**	C25H26FN5O3	4.24	0	9	
**CHEMBL484816**	C22H21N7O2	4.3	0	9	
**CHEMBL3218891**	C27H32N6O3	4.37	0	9	
**CHEMBL2158050**	C25H29FN2O4S2	4.63	0	9	
**CHEMBL3650850**	C21H23N5O4	4.68	0	9	
**CHEMBL4084170**	C15H18F5N3S	4.69	0	9	
**CHEMBL3612928**	C24H28N4O5	4.7	0	9	
**CHEMBL3422244**	C18H18F3N3O2S	4.71	0	9	
**CHEMBL3786311**	C26H30N4O5	4.8	0	9	

Table S4 (cont.d)

CHEMBL596700	C20H23F3N4O2	4.88	0	9	
**CHEMBL1083118**	C17H19F2N3O3S	4.89	0	9	
**CHEMBL2441417**	C24H26N6O3	4.89	0	9	
**CHEMBL3416021**	C20H24N8O	5.07	0	9	
**CHEMBL502288**	C23H22N6O2S	5.11	0	9	
**CHEMBL3612926**	C25H30N4O5	5.11	0	9	
**CHEMBL2164393**	C26H24F3N3O3	5.14	0	9	
**CHEMBL2170611**	C17H21F3N4O2	5.16	0	9	
**CHEMBL402015**	C27H27BrN6OS	5.2	0	9	
**CHEMBL429458**	C25H30FN3O5	5.2	0	9	
**CHEMBL4075071**	C19H16F3N5O	5.25	0	9	
**CHEMBL4243499**	C19H16F3N5O	5.25	0	9	
**CHEMBL2164389**	C24H20F3N3O3	5.34	0	9	
**CHEMBL1927161**	C24H29N5O4	5.4	0	9	
**CHEMBL244083**	C29H36N4O4S	5.6	0	9	
**CHEMBL245119**	C25H31N7OS	5.6	0	9	
**CHEMBL4213486**	C19H15F2N5O2	5.7	0	9	
**CHEMBL429761**	C23H31N7O2	5.73	0	9	
**CHEMBL3318999**	C28H25N7O2	5.85	0	9	
**CHEMBL446966**	C34H31N7O2	5.91	0	9	
**CHEMBL550410**	C25H26F4N4O	5.92	0	9	
**CHEMBL514042**	C25H38N6O3	6.05	0	9	
**CHEMBL549635**	C29H33F4N3O2	6.2	0	9	
**CHEMBL217442**	C24H23FN2O6	7.8	0	9	
**CHEMBL387178**	C23H20F2N2O5	8.21	0	9	
**CHEMBL3422973**	C27H28N6O3	8.68	0	9	
**CHEMBL3422970**	C27H28N6O3	9.06	0	9	
**CHEMBL3921669**	C23H26N4O5S	2.22	0	10	5.27
**CHEMBL2424928**	C24H26FN5O4	3.51	0	10	
**CHEMBL1916543**	C23H28N6O4	3.69	0	10	

Table S4 (cont.d)

CHEMBL605785	C27H27FN4O5	4.02	0	10	
**CHEMBL4112037**	C16H14F2N6OS	4.16	0	10	
**CHEMBL2151322**	C19H20FN7O2	4.28	0	10	
**CHEMBL3794265**	C23H25FN6O3	4.38	0	10	
**CHEMBL247690**	C29H33F2N3O5	4.47	0	10	
**CHEMBL3425929**	C28H33FN6O3	4.51	0	10	
**CHEMBL2041188**	C32H30N6O4	4.64	0	10	
**CHEMBL2324520**	C20H20F2N6O2	4.68	0	10	
**CHEMBL2315921**	C26H28F3N5O2	4.77	0	10	
**CHEMBL563791**	C28H29F3N6O	4.9	0	10	
**CHEMBL3400819**	C26H26FN5O4	4.91	0	10	
**CHEMBL595944**	C27H26N8O2	4.98	0	10	
**CHEMBL2164365**	C23H19F3N4O3	5.04	0	10	
**CHEMBL2177736**	C19H23N9O	5.07	0	10	
**CHEMBL3681314**	C26H25F2N7O	5.07	0	10	
**CHEMBL513921**	C23H34N6O4	5.24	0	10	
**CHEMBL3408394**	C37H24N6O2S2	5.24	0	10	
**CHEMBL562285**	C24H27N3O2	5.27	0	10	
**CHEMBL2164047**	C24H19F4N3O3	5.28	0	10	
**CHEMBL245642**	C25H25F2N3O5	5.5	0	10	
**CHEMBL551281**	C26H34F3N5O2	5.52	0	10	
**CHEMBL1236904**	C26H31BrN8O	5.52	0	10	
**CHEMBL3604800**	C28H32FN5O4	5.52	0	10	
**CHEMBL452823**	C37H38N10	5.67	0	10	
**CHEMBL553196**	C32H32F4N4O2	5.76	0	10	
**CHEMBL3323074**	C32H28FN7O2	5.85	0	10	
**CHEMBL3323073**	C32H28FN7O2	6.2	0	10	
**CHEMBL3605145**	C29H32FN5O4	6.3	0	10	
**CHEMBL1081867**	C22H23F3N6S	6.33	0	10	
**CHEMBL3605140**	C27H30FN5O4	6.4	0	10	

Table S4 (cont.d)

CHEMBL1081746	C23H26F3N5S2	6.46	0	10	
**CHEMBL401576**	C25H26F3N5OS	6.9	0	10	
**CHEMBL4081080**	C25H17F6N3O	7.17	0	10	
**CHEMBL390649**	C28H31F3N6S	7.5	0	10	
**CHEMBL428594**	C29H33F7N2O	7.8	0	10	
**CHEMBL217707**	C23H19F3N2O5	8.25	0	10	
**CHEMBL473**	C19H27N3O5S2	8.39	0	10	
**CHEMBL213715**	C24H22F2N2O6	8.44	0	10	
**CHEMBL3985847**	C21H26N6O4S	2.40	0	11	5
**CHEMBL1782574**	C23H35F3N4O3S	2.41	0	11	
**CHEMBL3964789**	C21H22F4N6O	3.17	0	11	
**CHEMBL3400817**	C26H26FN5O5	3.76	0	11	
**CHEMBL2165057**	C25H30N6O5	4.06	0	11	
**CHEMBL1916544**	C22H27N7O4	4.09	0	11	
**CHEMBL3703025**	C22H25F3N6O2	4.5	0	11	
**CHEMBL1091605**	C29H36F3N3O4S	4.52	0	11	
**CHEMBL1091218**	C29H25F3N2O6	4.58	0	11	
**CHEMBL2069410**	C23H19F4N3O4	4.6	0	11	
**CHEMBL2204260**	C22H25F3N4O3S	4.62	0	11	
**CHEMBL4209441**	C26H23FN8O2	4.62	0	11	
**CHEMBL4211893**	C30H29FN8O2	4.87	0	11	
**CHEMBL2164375**	C25H26F3N3O5	4.96	0	11	
**CHEMBL2204270**	C24H26F4N4O3	5	0	11	
**CHEMBL3318984**	C23H23F3N6O2	5	0	11	
**CHEMBL508098**	C36H37N11	5.08	0	11	
**CHEMBL3746204**	C22H17F4N5O2	5.24	0	11	
**CHEMBL2331648**	C20H22F3N5O3	5.34	0	11	
**CHEMBL4075908**	C20H21BrFN5O3S	5.46	0	11	
**CHEMBL192**	C22H30N6O4S	5.48	0	11	
**CHEMBL3422758**	C27H29F2N5O4	5.76	0	11	

Table S4 (cont.d)

**CHEMBL2041175**	**C31H27N7O3S**	**5.82**	**0**	**11**	
**CHEMBL256154**	C27H32F6N4O	5.85	0	11	
**CHEMBL1082111**	C22H23F4N5OS	5.9	0	11	
**CHEMBL402016**	C28H27F3N6OS	6	0	11	
**CHEMBL3605123**	C30H36FN5O5	6.16	0	11	
**CHEMBL411293**	C28H28F3N7S	6.6	0	11	
**CHEMBL3422952**	C26H27F2N5O4	9.14	0	11	
**CHEMBL3422978**	C26H26F3N5O3	9.41	0	11	
**CHEMBL491571**	C15H24N4O6S2	2.1	0	12	4.59
**CHEMBL254316**	C20H21FN6O5	2.5	0	12	
**CHEMBL3425799**	C26H25F2N5O5	3.48	0	12	
**CHEMBL1784523**	C36H40F3N5O3S	4	0	12	
**CHEMBL3417745**	C23H25F3N2O6S	4	0	12	
**CHEMBL451887**	C40H57N5O7	4.04	0	12	
**CHEMBL3342693**	C23H22F5N5O2	4.08	0	12	
**CHEMBL3425807**	C25H25FN6O5	4.52	0	12	
**CHEMBL2206791**	C23H21F3N6O3	4.58	0	12	
**CHEMBL2147316**	C22H19F5N4O3	4.6	0	12	
**CHEMBL2336323**	C26H30F2N6O4	4.62	0	12	
**CHEMBL2147303**	C22H19F5N4O3	4.7	0	12	
**CHEMBL402624**	C30H34F6N2O4	4.71	0	12	
**CHEMBL3605131**	C29H32FN5O6	4.91	0	12	
**CHEMBL3288030**	C44H53FN6O5	5.2	0	12	
**CHEMBL2441412**	C24H23F3N6O3	5.29	0	12	
**CHEMBL550535**	C27H33F6N5O	5.41	0	12	
**CHEMBL3605006**	C31H34FN5O6	5.8	0	12	
**CHEMBL195378**	C23H24F4N6O3	4.23	0	13	5.29
**CHEMBL3605022**	C29H32FN7O5	4.54	0	13	
**CHEMBL370072**	C24H24F4N4O5	4.55	0	13	
**CHEMBL3218816**	C19H17F3N8O2	4.75	0	13	

Table S4 (cont.d)

CHEMBL1099069	C19H24F3N9O	5.29	0	13	
**CHEMBL4079820**	C26H23F4N7O2	5.62	0	13	
**CHEMBL490026**	C24H24F6N2O4S	6.05	0	13	
**CHEMBL4077588**	C29H29F4N7O2	7	0	13	
**CHEMBL3422957**	C27H27F4N5O4	8.77	0	13	
**CHEMBL231396**	C20H17F6N5O3	4	0	14	
**CHEMBL4097247**	C27H26F3N7O4	5.24	0	14	
**CHEMBL435882**	C25H26F7N3O4	6.06	0	14	
**CHEMBL487708**	C25H22F6N4O3S	6.3	0	14	
**CHEMBL1908360**	C53H83NO14	3.3	0	15	
**CHEMBL3393730**	C42H52N8O6S2	4.6	0	16	
**CHEMBL1684059**	C20H28ClN	5.37	1	2	
**CHEMBL3314420**	C15H13ClFN	4.3	1	3	5.6
**CHEMBL1945694**	C16H15ClN2	5.16	1	3	
**CHEMBL191054**	C13H17ClN2	5.6	1	3	
**CHEMBL299390**	C19H19ClFN	5.83	1	3	
**CHEMBL505**	C16H19ClN2	5.96	1	3	
**CHEMBL1083407**	C21H22ClN3	5.44	1	4	
**CHEMBL53866**	C17H15ClFNO	5.66	1	4	
**CHEMBL3696120**	C22H26ClN3	5.96	1	4	
**CHEMBL384400**	C27H36ClN3O	5.48	1	5	6.72
**CHEMBL562833**	C22H30ClN3O	6.1	1	5	
**CHEMBL42**	C18H19ClN4	6.72	1	5	
**CHEMBL998**	C22H23ClN2O2	6.77	1	5	
**CHEMBL195180**	C24H27ClN2OS	7.52	1	5	
**CHEMBL4787638**	C21H18ClN3O2	2.08	1	6	5.05
**CHEMBL1688**	C18H19ClN4O	3.85	1	6	
**CHEMBL1000**	C21H25ClN2O3	4.52	1	6	
**CHEMBL494406**	C21H24ClN5	5.05	1	6	
**CHEMBL2146805**	C20H21ClN4O	5.6	1	6	

Table S4 (cont.d)

CHEMBL563926	C21H26ClN3O2	6.2	1	6	
**CHEMBL2146810**	C23H27ClN4O	8.4	1	6	
**CHEMBL4781551**	C24H21ClN4O2	4.08	1	7	5.45
**CHEMBL4127458**	C22H22ClN5O	4.22	1	7	
**CHEMBL512975**	C21H23ClFN5	4.64	1	7	
**CHEMBL2333616**	C20H22ClF3N2O	4.92	1	7	
**CHEMBL1957792**	C27H30ClN3O3	4.99	1	7	
**CHEMBL1092106**	C20H24ClFN4O	5.04	1	7	
**CHEMBL1091834**	C25H26ClFN4O	5.42	1	7	
**CHEMBL250533**	C25H28ClN3O3	5.48	1	7	
**CHEMBL4102707**	C20H20ClF4NO	5.54	1	7	
**CHEMBL3086038**	C30H33BrClN3O2	5.82	1	7	
**CHEMBL2146812**	C19H20ClN5O	5.9	1	7	
**CHEMBL3806153**	C18H21ClFN3O2	6.01	1	7	
**CHEMBL708**	C21H21ClN4OS	6.92	1	7	
**CHEMBL12713**	C24H26ClFN4O	8.57	1	7	
**CHEMBL2178221**	C28H29ClN4O3	4.82	1	8	5.8
**CHEMBL1093696**	C24H25ClFN5O	4.92	1	8	
**CHEMBL2325997**	C20H23ClN6O	5.14	1	8	
**CHEMBL506163**	C22H19ClFN5O	5.2	1	8	
**CHEMBL3218650**	C27H30ClN5O2	5.6	1	8	
**CHEMBL3703237**	C19H17ClN4O3	5.66	1	8	
**CHEMBL2387229**	C26H26ClFN2O4	5.8	1	8	
**CHEMBL1956991**	C23H26ClFN4O2	5.89	1	8	
**CHEMBL3318989**	C23H24ClN5O2	6	1	8	
**CHEMBL2146813**	C38H48ClN5O2	6.8	1	8	
**CHEMBL525413**	C29H24ClN5O2	7.09	1	8	
**CHEMBL244278**	C26H28ClN5OS	7.1	1	8	
**CHEMBL217789**	C23H21ClN2O5	8.44	1	8	
**CHEMBL3608687**	C16H15ClN6OS	4.18	1	9	5.68

Table S4 (cont.d)

CHEMBL4094682	C22H27ClN6O2	4.34	1	9	
**CHEMBL2325729**	C26H34ClN7O	4.53	1	9	
**CHEMBL3262358**	C21H22ClN7O	4.7	1	9	
**CHEMBL255968**	C25H24ClN5O3	5	1	9	
**CHEMBL1091664**	C28H25ClN2O5S	5	1	9	
**CHEMBL489324**	C28H42ClF2N5O	5.68	1	9	
**CHEMBL3237444**	C23H25ClN8	5.7	1	9	
**CHEMBL2177305**	C28H23ClFN5O2	5.89	1	9	
**CHEMBL557306**	C21H24ClF3N2O3	5.99	1	9	
**CHEMBL1081747**	C21H23ClFN5OS	6.4	1	9	
**CHEMBL3983509**	C20H20ClFN4O2S	6.7	1	9	
**CHEMBL1729**	C23H29ClFN3O4	8.19	1	9	
**CHEMBL2440387**	C25H23ClN4O4S	3.38	1	10	4.73
**CHEMBL3221500**	C22H21ClN6O3	4.2	1	10	
**CHEMBL3221488**	C23H23ClN6O3	4.5	1	10	
**CHEMBL256060**	C26H26ClN5O4	4.96	1	10	
**CHEMBL2403108**	C28H36ClN5O3S	5.9	1	10	
**CHEMBL375330**	C34H38ClF3N4O2	6.77	1	10	
**CHEMBL3221490**	C23H22ClN7O3	4.4	1	11	4.95
**CHEMBL3407784**	C20H19ClN6O3S	4.89	1	11	
**CHEMBL3221503**	C23H20ClN7O3	4.9	1	11	
**CHEMBL4076061**	C22H20ClF2N3O3S2	4.99	1	11	
**CHEMBL3353404**	C26H29ClN8O2	5.17	1	11	
**CHEMBL2147223**	C22H20ClF3N4O3	5.4	1	11	
**CHEMBL3608744**	C28H31ClN6O4S	4.09	1	12	
**CHEMBL3221487**	C20H19ClN8O3	4.8	1	12	
**CHEMBL2064657**	C28H23ClF2N6O3	4.91	1	12	
**CHEMBL401608**	C27H31ClF6N4O2	6.73	1	13	
**CHEMBL1684047**	C16H23Cl2N	4.4	2	3	
**CHEMBL2324239**	C24H18Cl2NO2^{+}	4.51	2	5	

Table S4 (cont.d)

CHEMBL1080790	C16H22Cl2N2O	4.78	2	5	
**CHEMBL1080969**	C16H22Cl2N2O	5.11	2	5	
**CHEMBL2324245**	$C24H26Cl2NO2^{+}$	7.22	2	5	
**CHEMBL3741589**	C18H17Cl2NO3	4.11	2	6	
**CHEMBL1083463**	C26H26Cl2N4	6.22	2	6	
**CHEMBL2158823**	C24H28Cl2N2O3	4.5	2	7	
**CHEMBL2158815**	C24H28Cl2N2O3	5.3	2	7	
**CHEMBL3806193**	C19H23Cl2N3O2	5.84	2	7	
**CHEMBL1107**	C26H30Cl2F3NO	7.4	2	7	
**CHEMBL540634**	C26H31Cl2N3O3	4.5	2	8	5.15
**CHEMBL3216126**	C25H35Cl2N5O	4.65	2	8	
**CHEMBL3964723**	C23H27Cl2N3O3	5	2	8	
**CHEMBL2158771**	C27H31Cl2N3O3	5.3	2	8	
**CHEMBL233047**	C20H32Cl2N6	6.52	2	8	
**CHEMBL128007**	C28H28Cl2N4O2	7	2	8	
**CHEMBL589833**	C24H25Cl2FN4O2	5.1	2	9	5.36
**CHEMBL4170966**	C24H21Cl2N3O4	5.33	2	9	
**CHEMBL255645**	C28H31Cl2N5O2	5.36	2	9	
**CHEMBL2158625**	C20H19Cl2N5OS	5.44	2	9	
**CHEMBL2158629**	C20H21Cl2N5OS	5.85	2	9	
**CHEMBL560063**	C24H23Cl2F3N4O	5.46	2	10	
**CHEMBL537847**	C23H29Cl2N5O3	6.25	2	10	
**CHEMBL3764895**	C29H31Cl2F3N4O3	5.13	2	12	
**CHEMBL3764357**	C29H31Cl2F3N4O3	5.2	2	12	
**CHEMBL2207664**	C22H24Cl2F3N3O4	5.3	2	12	
**CHEMBL3765012**	C30H31Cl2F3N4O3	5.51	2	12	
**CHEMBL1098847**	C24H21Cl3N2O2	5.58	3	7	
**CHEMBL1095476**	C28H25Cl3N4O2	5.06	3	9	
**CHEMBL498411**	C22H19Cl3N6	6.46	3	9	
**CHEMBL1097456**	C26H22Cl3F3N2O3	5.84	3	11	

Table S5. Investigation of the influence of oxygen on the pIC_{50} activity.

Name	Molecular formula	pIC50	O	Hetatoms	Median
**CHEMBL284348**	C5H6N2	2.36	0	2	5.11
**CHEMBL3**	C10H14N2	3.61	0	2	
**CHEMBL1086033**	C17H22N2	3.67	0	2	
**CHEMBL87045**	C18H18N2	5.06	0	2	
**CHEMBL459176**	C20H22N2	5.16	0	2	
**CHEMBL1684059**	C20H28ClN	5.37	0	2	
**CHEMBL11**	C19H24N2	5.47	0	2	
**CHEMBL1671894**	C27H26N2	7.75	0	2	
**CHEMBL3314420**	C15H13ClFN	4.30	0	3	5.80
**CHEMBL1684047**	C16H23Cl2N	4.40	0	3	
**CHEMBL513258**	C19H19FN2	5.05	0	3	
**CHEMBL1945694**	C16H15ClN2	5.16	0	3	
**CHEMBL191054**	C13H17ClN2	5.60	0	3	
**CHEMBL497171**	C15H17N3	5.80	0	3	
**CHEMBL299390**	C19H19ClFN	5.83	0	3	
**CHEMBL457930**	C20H19N3	5.85	0	3	
**CHEMBL505**	C16H19ClN2	5.96	0	3	
**CHEMBL1090528**	C19H24N2S	6.21	0	3	
**CHEMBL299499**	C17H22IN3	6.31	0	3	
**CHEMBL1671896**	C11H14F3N	3.27	0	4	5.70
**CHEMBL3314426**	C15H14F2N2	4.66	0	4	
**CHEMBL1083407**	C21H22ClN3	5.44	0	4	
**CHEMBL1093058**	C17H21N3S	5.70	0	4	
**CHEMBL3696120**	C22H26ClN3	5.96	0	4	
**CHEMBL1092377**	C19H21FN2S	6.48	0	4	
**CHEMBL2046892**	C22H22F2N2	7.40	0	4	
**CHEMBL1125**	C16H18N4S	4.90	0	5	5.33
**CHEMBL237191**	C25H31N5	4.98	0	5	
**CHEMBL2326478**	C18H18FN3S	5.17	0	5	
**CHEMBL1956112**	C21H28N4S	5.33	0	5	

Table S5 (Cont.d)

**CHEMBL42**	**C18H19ClN4**	**6.72**	**0**	**5**	
**CHEMBL715**	C17H20N4S	6.74	0	5	
**CHEMBL61301**	C19H21FN4	7.56	0	5	
**CHEMBL460491**	C21H34N4S2	4.88	0	6	5.80
**CHEMBL494406**	C21H24ClN5	5.05	0	6	
**CHEMBL2177904**	C23H16FN5	5.51	0	6	
**CHEMBL2177905**	C24H16F2N4	5.80	0	6	
**CHEMBL2147024**	C22H18N6	5.96	0	6	
**CHEMBL1083463**	C26H26Cl2N4	6.22	0	6	
**CHEMBL522456**	CHEMBL522456	7.00	0	6	
**CHEMBL474484**	C13H21N7	4.28	0	7	5.28
**CHEMBL512975**	C21H23ClFN5	4.64	0	7	
**CHEMBL575241**	C21H26N6S	4.71	0	7	
**CHEMBL523821**	C21H15F2N5	5.16	0	7	
**CHEMBL2178522**	C23H19FN6	5.28	0	7	
**CHEMBL2147022**	C20H17N7	5.37	0	7	
**CHEMBL2147021**	C20H15FN6	5.57	0	7	
**CHEMBL1084400**	C20H21F3N4	6.80	0	7	
**CHEMBL584766**	C23H24FN5S	7.00	0	7	
**CHEMBL640**	C13H21N3O	3.86	1	4	4.91
**CHEMBL219803**	C17H25N3O	3.94	1	4	
**CHEMBL219128**	C17H33N3O	4.17	1	4	
**CHEMBL219074**	C20H31N3O	4.42	1	4	
**CHEMBL1086273**	C19H23FN2O	4.68	1	4	
**CHEMBL1089159**	C22H23N3O	4.70	1	4	
**CHEMBL2333646**	C19H23BrN2O	4.89	1	4	
**CHEMBL517**	C21H29N3O	4.92	1	4	
**CHEMBL562948**	C22H33N3O	5.40	1	4	
**CHEMBL1090175**	C20H25N3O	5.47	1	4	
**CHEMBL53866**	C17H15ClFNO	5.66	1	4	

Table S5 (cont.d)

**CHEMBL1935443**	**C20H23N3O**	**5.68**	**1**	**4**	
**CHEMBL3323181**	C22H24N2OS	6.00	1	4	
**CHEMBL460893**	C23H25N3O	6.13	1	4	
**CHEMBL4213196**	C25H34N4O	4.62	1	5	5.20
**CHEMBL217133**	C18H22N4O	4.68	1	5	
**CHEMBL429115**	C20H24N4O	4.71	1	5	
**CHEMBL1080790**	C16H22Cl2N2O	4.78	1	5	
**CHEMBL4096683**	C20H22N4O	4.94	1	5	
**CHEMBL565829**	C15H14N4O	5.01	1	5	
**CHEMBL2177304**	C27H24FN3O	5.07	1	5	
**CHEMBL1080969**	C16H22Cl2N2O	5.11	1	5	
**CHEMBL4100591**	C27H34F2N2O	5.29	1	5	
**CHEMBL384400**	C27H36ClN3O	5.48	1	5	
**CHEMBL1110**	C17H18N4O	5.64	1	5	
**CHEMBL562833**	C22H30ClN3O	6.10	1	5	
**CHEMBL58387**	C31H38N4O	6.24	1	5	
**CHEMBL3775729**	C26H28N4O	6.28	1	5	
**CHEMBL1087493**	C17H26N4O	6.63	1	5	
**CHEMBL195180**	C24H27ClN2OS	7.52	1	5	
**CHEMBL1688**	C18H19ClN4O	3.85	1	6	5.96
**CHEMBL4212656**	C24H31N5O	4.64	1	6	
**CHEMBL398612**	C26H35FN4O	4.88	1	6	
**CHEMBL4067770**	C20H21FN4O	5.11	1	6	
**CHEMBL4100776**	C20H22N4OS	5.23	1	6	
**CHEMBL225036**	C24H26N4OS	5.29	1	6	
**CHEMBL2146805**	C20H21ClN4O	5.60	1	6	
**CHEMBL4104102**	C20H22N4OS	5.61	1	6	
**CHEMBL4081790**	C25H19N5O	6.30	1	6	
**CHEMBL4096145**	C22H27N5O	6.52	1	6	

Table S5 (cont.d)

CHEMBL3774817	C25H25FN4O	6.59	1	6	
**CHEMBL329067**	C32H33N5O	7.62	1	6	
**CHEMBL12780**	C24H27FN4O	8.16	1	6	
**CHEMBL2146810**	C23H27ClN4O	8.40	1	6	
**CHEMBL1423**	C28H29F2N3O	8.52	1	6	
**CHEMBL296419**	C28H31FN4O	9.05	1	6	
**CHEMBL578834**	C21H25N5OS	3.72	1	7	5.07
**CHEMBL3582294**	C25H18F2N4O	4.03	1	7	
**CHEMBL4224807**	C14H16N6O	4.03	1	7	
**CHEMBL4127458**	C22H22ClN5O	4.22	1	7	
**CHEMBL218836**	C18H24F3N3O	4.40	1	7	
**CHEMBL2069925**	C21H21N5OS	4.52	1	7	
**CHEMBL2333636**	C24H28F4N2O	4.75	1	7	
**CHEMBL2180842**	C17H22FN5O	4.80	1	7	
**CHEMBL2314058**	C25H34F3N3O	4.83	1	7	
**CHEMBL2333616**	C20H22ClF3N2O	4.92	1	7	
**CHEMBL1092106**	C20H24ClFN4O	5.04	1	7	
**CHEMBL2333607**	C25H30F4N2O	5.05	1	7	
**CHEMBL2333615**	C23H26F4N2O	5.05	1	7	
**CHEMBL3262625**	C23H26N6O	5.09	1	7	
**CHEMBL1091834**	C25H26ClFN4O	5.42	1	7	
**CHEMBL1813015**	C20H20N6O	5.42	1	7	
**CHEMBL4102707**	C20H20ClF4NO	5.54	1	7	
**CHEMBL2151218**	C25H22FN5O	5.70	1	7	
**CHEMBL243901**	C23H31N5OS	5.90	1	7	
**CHEMBL2146812**	C19H20ClN5O	5.90	1	7	
**CHEMBL1079578**	C28H31N5OS	6.20	1	7	
**CHEMBL3775050**	C25H24F2N4O	6.20	1	7	
**CHEMBL708**	C21H21ClN4OS	6.92	1	7	
**CHEMBL1107**	C26H30Cl2F3NO	7.40	1	7	
**CHEMBL4279819**	C16H18N6O	8.07	1	7	

Table S5 (cont.d)

CHEMBL12713	C24H26ClFN4O	8.57	1	7	
**CHEMBL3804950**	C20H21F2N5O	4.01	1	8	5.14
**CHEMBL4858551**	C19H19FN6O	4.01	1	8	
**CHEMBL485123**	C20H23F3N4O	4.07	1	8	
**CHEMBL2333619**	C22H27F4N3O	4.10	1	8	
**CHEMBL2180070**	C19H23N7O	4.27	1	8	
**CHEMBL256653**	C23H26N6OS	4.30	1	8	
**CHEMBL3216126**	C25H35Cl2N5O	4.65	1	8	
**CHEMBL3593771**	C19H19N7O	4.87	1	8	
**CHEMBL257901**	C28H30N6OS	4.90	1	8	
**CHEMBL270239**	C30H34N6OS	4.90	1	8	
**CHEMBL1093696**	C24H25ClFN5O	4.92	1	8	
**CHEMBL515001**	C21H25FN6O	4.96	1	8	
**CHEMBL2325997**	C20H23ClN6O	5.14	1	8	
**CHEMBL3287218**	C29H32N6OS	5.16	1	8	
**CHEMBL506163**	C22H19ClFN5O	5.20	1	8	
**CHEMBL262341**	C33H29N7O	5.25	1	8	
**CHEMBL270852**	C32H38N6OS	5.40	1	8	
**CHEMBL272086**	C32H38N6OS	5.60	1	8	
**CHEMBL399525**	C19H23N7O	5.75	1	8	
**CHEMBL2178521**	C24H21FN6O	5.75	1	8	
**CHEMBL408169**	C28H32N6OS	6.40	1	8	
**CHEMBL94454**	C24H25FN6O	6.46	1	8	
**CHEMBL244280**	C26H28FN5OS	6.60	1	8	
**CHEMBL244278**	C26H28ClN5OS	7.10	1	8	
**CHEMBL397429**	C28H30N6OS	7.30	1	8	
**CHEMBL3608687**	C16H15ClN6OS	4.18	1	9	5.35
**CHEMBL2325729**	C26H34ClN7O	4.53	1	9	
**CHEMBL3262358**	C21H22ClN7O	4.70	1	9	
**CHEMBL3416021**	C20H24N8O	5.07	1	9	
**CHEMBL402015**	C27H27BrN6OS	5.20	1	9	

Table S5 (cont.d)

CHEMBL4075071	C19H16F3N5O	5.25	1	9	
**CHEMBL2158625**	C20H19Cl2N5OS	5.44	1	9	
**CHEMBL245119**	C25H31N7OS	5.60	1	9	
**CHEMBL489324**	C28H42ClF2N5O	5.68	1	9	
**CHEMBL2158629**	C20H21Cl2N5OS	5.85	1	9	
**CHEMBL550410**	C25H26F4N4O	5.92	1	9	
**CHEMBL1081747**	C21H23ClFN5OS	6.40	1	9	
**CHEMBL4112037**	C16H14F2N6OS	4.16	1	10	5.37
**CHEMBL563791**	C28H29F3N6O	4.90	1	10	
**CHEMBL2177736**	C19H23N9O	5.07	1	10	
**CHEMBL3681314**	C26H25F2N7O	5.07	1	10	
**CHEMBL562285**	C24H27N3O2	5.27	1	10	
**CHEMBL560063**	C24H23Cl2F3N4O	5.46	1	10	
**CHEMBL1236904**	C26H31BrN8O	5.52	1	10	
**CHEMBL401576**	C25H26F3N5OS	6.90	1	10	
**CHEMBL4081080**	C25H17F6N3O	7.17	1	10	
**CHEMBL428594**	C29H33F7N2O	7.80	1	10	
**CHEMBL16**	C15H12N2O2	4.00	2	4	5.92
**CHEMBL3356251**	C24H36N2O2	4.01	2	4	
**CHEMBL3786542**	C26H26N2O2	4.21	2	4	
**CHEMBL3356249**	C25H38N2O2	4.30	2	4	
**CHEMBL3185736**	C22H20N2O2	4.91	2	4	
**CHEMBL4162513**	C35H34N2O2	5.10	2	4	
**CHEMBL4450665**	C23H28N2O2	5.92	2	4	
**CHEMBL1346**	C28H30N2O2	7.10	2	4	
**CHEMBL1223951**	C21H24N2O2	9.21	2	4	
**CHEMBL1257938**	C22H26N2O2	9.21	2	4	
**CHEMBL1257937**	C22H26N2O2	9.28	2	4	
**CHEMBL1257820**	C23H28N2O2	9.60	2	4	
**CHEMBL1257821**	C22H26N2O2	9.85	2	4	
**CHEMBL1097**	C15H23N3O2	4.00	2	5	5.18

Table S5 (cont.d)

CHEMBL4209354	C15H20N2O2S	4.07	2	5	
**CHEMBL1091879**	C19H25N3O2	4.12	2	5	
**CHEMBL561138**	C23H27N3O2	4.14	2	5	
**CHEMBL1258006**	C22H23N3O2	4.27	2	5	
**CHEMBL2324239**	C24H18Cl2NO2^{+}	4.51	2	5	
**CHEMBL1094041**	C29H29N3O2	4.55	2	5	
**CHEMBL2441636**	C22H33N3O2	4.72	2	5	
**CHEMBL487063**	C23H27N3O2	5.10	2	5	
**CHEMBL378547**	C21H21F2NO2	5.11	2	5	
**CHEMBL485242**	C25H29N3O2	5.14	2	5	
**CHEMBL478615**	C23H27N3O2	5.17	2	5	
**CHEMBL476579**	C24H29N3O2	5.18	2	5	
**CHEMBL479242**	C23H27N3O2	5.18	2	5	
**CHEMBL3121096**	C20H25N3O2	5.22	2	5	
**CHEMBL1819137**	C22H25N3O2	5.23	2	5	
**CHEMBL488249**	C24H27N3O2	5.25	2	5	
**CHEMBL487064**	C23H27N3O2	5.55	2	5	
**CHEMBL478616**	C23H27N3O2	5.80	2	5	
**CHEMBL1224697**	C22H28N2O2S	5.90	2	5	
**CHEMBL376488**	C32H31BrN2O2	6.43	2	5	
**CHEMBL998**	C22H23ClN2O2	6.77	2	5	
**CHEMBL2324245**	C24H26Cl2NO2^{+}	7.22	2	5	
**CHEMBL259732**	C20H27N3O2	9.19	2	5	
**CHEMBL1257577**	C19H22N2O2S	9.37	2	5	
**CHEMBL410832**	C23H25N3O2	9.39	2	5	
**CHEMBL1257578**	C20H24N2O2S	9.59	2	5	
**CHEMBL4787638**	C21H18ClN3O2	2.08	2	6	5.55
**CHEMBL1823042**	C20H28FN3O2	5.00	2	6	
**CHEMBL4089699**	C19H19N3O2S	5.42	2	6	
**CHEMBL1823047**	C22H30N4O2	5.54	2	6	
**CHEMBL4065208**	C23H22N4O2	5.56	2	6	

Table S5 (cont.d)

CHEMBL563926	C21H26ClN3O2	6.20	2	6	
**CHEMBL3775282**	C26H28N4O2	7.16	2	6	
**CHEMBL1108**	C22H22FN3O2	7.49	2	6	
**CHEMBL4781551**	C24H21ClN4O2	4.08	2	7	5.42
**CHEMBL1957012**	C26H29N5O2	4.60	2	7	
**CHEMBL3973288**	C20H22FN3O2S	4.84	2	7	
**CHEMBL523374**	C22H23N5O2	4.89	2	7	
**CHEMBL3218884**	C28H33N5O2	4.96	2	7	
**CHEMBL498042**	C24H23N5O2	5.08	2	7	
**CHEMBL497048**	C27H23N5O2	5.36	2	7	
**CHEMBL2336331**	C24H29N5O2	5.40	2	7	
**CHEMBL1939739**	C32H35FN4O2	5.44	2	7	
**CHEMBL1098847**	C24H21Cl3N2O2	5.58	2	7	
**CHEMBL3086038**	C30H33BrClN3O2	5.82	2	7	
**CHEMBL3806193**	C19H23Cl2N3O2	5.84	2	7	
**CHEMBL3775387**	C25H27N5O2	5.99	2	7	
**CHEMBL3806153**	C18H21ClFN3O2	6.01	2	7	
**CHEMBL501480**	C29H25N5O2	6.50	2	7	
**CHEMBL526466**	C25H23N5O2	7.10	2	7	
**CHEMBL399726**	C20H24N6O2	3.78	2	8	5.84
**CHEMBL2314064**	C24H32F3N3O2	4.41	2	8	
**CHEMBL3577935**	C23H34F3N3O2	4.50	2	8	
**CHEMBL2164565**	C19H18F3N3O2	4.63	2	8	
**CHEMBL1939742**	C30H37N5O2S	4.75	2	8	
**CHEMBL2181489**	C23H20F3N3O2	5.52	2	8	
**CHEMBL3218650**	C27H30ClN5O2	5.60	2	8	
**CHEMBL3415593**	C19H26N6O2	5.77	2	8	
**CHEMBL1080489**	C22H27N5O2S	5.80	2	8	
**CHEMBL478462**	C25H38N6O2	5.84	2	8	
**CHEMBL1956991**	C23H26ClFN4O2	5.89	2	8	
**CHEMBL3318989**	C23H24ClN5O2	6.00	2	8	

Table S5 (cont.d)

**CHEMBL515025**	**C24H36N6O2**	**6.15**	**2**	**8**	
**CHEMBL478011**	C25H38N6O2	6.38	2	8	
**CHEMBL453894**	C25H26F3N3O2	6.46	2	8	
**CHEMBL2146813**	C38H48ClN5O2	6.80	2	8	
**CHEMBL568571**	C27H26F3N3O2	6.85	2	8	
**CHEMBL128007**	C28H28Cl2N4O2	7.00	2	8	
**CHEMBL525413**	C29H24ClN5O2	7.09	2	8	
**CHEMBL2324519**	C20H21FN6O2	3.67	2	9	5.14
**CHEMBL3290351**	C24H27FN6O2	4.05	2	9	
**CHEMBL484816**	C22H21N7O2	4.30	2	9	
**CHEMBL4094682**	C22H27ClN6O2	4.34	2	9	
**CHEMBL3422244**	C18H18F3N3O2S	4.71	2	9	
**CHEMBL596700**	C20H23F3N4O2	4.88	2	9	
**CHEMBL1095476**	C28H25Cl3N4O2	5.06	2	9	
**CHEMBL589833**	C24H25Cl2FN4O2	5.10	2	9	
**CHEMBL502288**	C23H22N6O2S	5.11	2	9	
**CHEMBL2170611**	C17H21F3N4O2	5.16	2	9	
**CHEMBL255645**	C28H31Cl2N5O2	5.36	2	9	
**CHEMBL4213486**	C19H15F2N5O2	5.70	2	9	
**CHEMBL429761**	C23H31N7O2	5.73	2	9	
**CHEMBL3318999**	C28H25N7O2	5.85	2	9	
**CHEMBL2177305**	C28H23ClFN5O2	5.89	2	9	
**CHEMBL446966**	C34H31N7O2	5.91	2	9	
**CHEMBL549635**	C29H33F4N3O2	6.20	2	9	
**CHEMBL3983509**	C20H20ClFN4O2S	6.70	2	9	
**CHEMBL2151322**	C19H20FN7O2	4.28	2	10	5.38
**CHEMBL2324520**	C20H20F2N6O2	4.68	2	10	
**CHEMBL2315921**	C26H28F3N5O2	4.77	2	10	
**CHEMBL595944**	C27H26N8O2	4.98	2	10	
**CHEMBL3408394**	C37H24N6O2S2	5.24	2	10	
**CHEMBL551281**	C26H34F3N5O2	5.52	2	10	

Table S5 (cont.d)

**CHEMBL553196**	**C32H32F4N4O2**	**5.76**	**2**	**10**	
**CHEMBL3323074**	C32H28FN7O2	5.85	2	10	
**CHEMBL3323073**	C32H28FN7O2	6.20	2	10	
**CHEMBL375330**	C34H38ClF3N4O2	6.77	2	10	
**CHEMBL3703025**	C22H25F3N6O2	4.50	2	11	4.94
**CHEMBL4209441**	C26H23FN8O2	4.62	2	11	
**CHEMBL4211893**	C30H29FN8O2	4.87	2	11	
**CHEMBL3318984**	C23H23F3N6O2	5.00	2	11	
**CHEMBL3353404**	C26H29ClN8O2	5.17	2	11	
**CHEMBL3746204**	C22H17F4N5O2	5.24	2	11	
**CHEMBL4640366**	C33H34N2O3	4.02	3	5	5.92
**CHEMBL3787069**	C26H26N2O3	4.35	3	5	
**CHEMBL498572**	C19H22N2O3	5.23	3	5	
**CHEMBL1671893**	C23H30N2O3	6.60	3	5	
**CHEMBL1642487**	C28H28N2O3	9.09	3	5	
**CHEMBL1642479**	C28H28N2O3	9.17	3	5	
**CHEMBL3741589**	C18H17Cl2NO3	4.11	3	6	5.92
**CHEMBL1000**	C21H25ClN2O3	4.52	3	6	
**CHEMBL3219616**	C24H30N2O3S	5.80	3	6	
**CHEMBL1224699**	C21H26N2O3S	5.90	3	6	
**CHEMBL561279**	C25H34N2O3S	5.93	3	6	
**CHEMBL4173253**	C26H34N2O3S	7.10	3	6	
**CHEMBL533**	C20H36N2O3S	8.00	3	6	
**CHEMBL1258503**	C22H25N3O3	9.14	3	6	
**CHEMBL8**	C17H18FN3O3	3.02	3	7	4.99
**CHEMBL4855895**	C21H24FN3O3	3.53	3	7	
**CHEMBL22**	C14H18N4O3	3.62	3	7	
**CHEMBL388978**	C28H26N4O3	4.04	3	7	
**CHEMBL583**	C19H22FN3O3	4.30	3	7	
**CHEMBL2158823**	C24H28Cl2N2O3	4.50	3	7	
**CHEMBL3115194**	C26H32BrN3O3	4.90	3	7	

Table S5 (cont.d)

**CHEMBL1957792**	**C27H30ClN3O3**	**4.99**	**3**	**7**	
**CHEMBL246815**	C22H26FN3O3	5.13	3	7	
**CHEMBL398478**	C25H28FN3O3	5.21	3	7	
**CHEMBL2158815**	C24H28Cl2N2O3	5.30	3	7	
**CHEMBL250533**	C25H28ClN3O3	5.48	3	7	
**CHEMBL3764774**	C28H28N4O3	5.70	3	7	
**CHEMBL45816**	C29H38FN3O3	6.24	3	7	
**CHEMBL2146854**	C23H26N4O3	7.33	3	7	
**CHEMBL540634**	C26H31Cl2N3O3	4.50	3	8	5.35
**CHEMBL2178221**	C28H29ClN4O3	4.82	3	8	
**CHEMBL3964723**	C23H27Cl2N3O3	5.00	3	8	
**CHEMBL465417**	C25H23N5O3	5.04	3	8	
**CHEMBL2158771**	C27H31Cl2N3O3	5.30	3	8	
**CHEMBL207220**	C24H40N4O3S	5.40	3	8	
**CHEMBL2207738**	C23H26N4O3S	5.60	3	8	
**CHEMBL3703237**	C19H17ClN4O3	5.66	3	8	
**CHEMBL1621**	C23H27FN4O3	6.00	3	8	
**CHEMBL2146853**	C20H23N5O3	7.60	3	8	
**CHEMBL3290344**	C24H26N6O3	4.13	3	9	5.07
**CHEMBL2165068**	C25H26FN5O3	4.24	3	9	
**CHEMBL3218891**	C27H32N6O3	4.37	3	9	
**CHEMBL1083118**	C17H19F2N3O3S	4.89	3	9	
**CHEMBL2441417**	C24H26N6O3	4.89	3	9	
**CHEMBL255968**	C25H24ClN5O3	5.00	3	9	
**CHEMBL2164393**	C26H24F3N3O3	5.14	3	9	
**CHEMBL2164389**	C24H20F3N3O3	5.34	3	9	
**CHEMBL557306**	C21H24ClF3N2O3	5.99	3	9	
**CHEMBL514042**	C25H38N6O3	6.05	3	9	
**CHEMBL3422973**	C27H28N6O3	8.68	3	9	
**CHEMBL3422970**	C27H28N6O3	9.06	3	9	
**CHEMBL3221500**	C22H21ClN6O3	4.20	3	10	4.78

Table S5 (cont.d)

**CHEMBL3794265**	**C23H25FN6O3**	**4.38**	**3**	**10**	
**CHEMBL3221488**	C23H23ClN6O3	4.50	3	10	
**CHEMBL3425929**	C28H33FN6O3	4.51	3	10	
**CHEMBL2164365**	C23H19F3N4O3	5.04	3	10	
**CHEMBL2164047**	C24H19F4N3O3	5.28	3	10	
**CHEMBL2403108**	C28H36ClN5O3S	5.90	3	10	
**CHEMBL537847**	C23H29Cl2N5O3	6.25	3	10	
**CHEMBL1782574**	C23H35F3N4O3S	2.41	3	11	5.00
**CHEMBL3221490**	C23H22ClN7O3	4.40	3	11	
**CHEMBL2204260**	C22H25F3N4O3S	4.62	3	11	
**CHEMBL3407784**	C20H19ClN6O3S	4.89	3	11	
**CHEMBL3221503**	C23H20ClN7O3	4.90	3	11	
**CHEMBL4076061**	C22H20ClF2N3O3S2	4.99	3	11	
**CHEMBL2204270**	C24H26F4N4O3	5.00	3	11	
**CHEMBL2331648**	C20H22F3N5O3	5.34	3	11	
**CHEMBL2147223**	C22H20ClF3N4O3	5.40	3	11	
**CHEMBL4075908**	C20H21BrFN5O3S	5.46	3	11	
**CHEMBL2041175**	C31H27N7O3S	5.82	3	11	
**CHEMBL1097456**	C26H22Cl3F3N2O3	5.84	3	11	
**CHEMBL3422978**	C26H26F3N5O3	9.41	3	11	
**CHEMBL1784523**	C36H40F3N5O3S	4.00	3	12	4.86
**CHEMBL2206791**	C23H21F3N6O3	4.58	3	12	
**CHEMBL2147316**	C22H19F5N4O3	4.60	3	12	
**CHEMBL2147303**	C22H19F5N4O3	4.70	3	12	
**CHEMBL3221487**	C20H19ClN8O3	4.80	3	12	
**CHEMBL2064657**	C28H23ClF2N6O3	4.91	3	12	
**CHEMBL3764895**	C29H31Cl2F3N4O3	5.13	3	12	
**CHEMBL3764357**	C29H31Cl2F3N4O3	5.20	3	12	
**CHEMBL2441412**	C24H23F3N6O3	5.29	3	12	
**CHEMBL3765012**	C30H31Cl2F3N4O3	5.51	3	12	
**CHEMBL270190**	C25H32N2O4	3.10	4	6	5.88

Table S5 (cont.d)

CHEMBL519266	C22H28N2O4	3.11	4	6	
**CHEMBL1938433**	C23H24N2O4	5.42	4	6	
**CHEMBL556100**	C22H25NO4S	6.33	4	6	
**CHEMBL723**	C24H26N2O4	6.46	4	6	
**CHEMBL1258280**	C22H24N2O4	9.21	4	6	
**CHEMBL4847738**	C20H23N3O4	4.05	4	7	5.96
**CHEMBL3703271**	C22H23N3O4	5.08	4	7	
**CHEMBL246634**	C21H23FN2O4	5.58	4	7	
**CHEMBL17423**	C22H25N3O4	5.96	4	7	
**CHEMBL2387265**	C31H29FN2O4	6.09	4	7	
**CHEMBL384487**	C24H23FN2O4	7.69	4	7	
**CHEMBL217593**	C23H23FN2O4	8.27	4	7	
**CHEMBL33**	C18H20FN3O4	3.04	4	8	4.51
**CHEMBL31**	C19H22FN3O4	3.89	4	8	
**CHEMBL520463**	C28H24N4O4	4.05	4	8	
**CHEMBL32**	C21H24FN3O4	4.10	4	8	
**CHEMBL2382343**	C29H32F2N2O4	4.32	4	8	
**CHEMBL3787345**	C25H28N4O4	4.51	4	8	
**CHEMBL602875**	C26H34N4O4	4.72	4	8	
**CHEMBL3612814**	C24H28N4O4	4.85	4	8	
**CHEMBL1084617**	C24H28N4O4	4.96	4	8	
**CHEMBL3786346**	C25H26N4O4	5.05	4	8	
**CHEMBL2387229**	C26H26ClFN2O4	5.80	4	8	
**CHEMBL4092041**	C29H31F3N2O4	4.07	4	9	5.25
**CHEMBL248296**	C27H31F2N3O4	4.19	4	9	
**CHEMBL2158050**	C25H29FN2O4S2	4.63	4	9	
**CHEMBL3650850**	C21H23N5O4	4.68	4	9	
**CHEMBL4243499**	C19H16F3N5O	5.25	4	9	
**CHEMBL4170966**	C24H21Cl2N3O4	5.33	4	9	
**CHEMBL1927161**	C24H29N5O4	5.40	4	9	
**CHEMBL244083**	C29H36N4O4S	5.60	4	9	

Table S5 (cont.d)

**CHEMBL1729**	**C23H29ClFN3O4**	**8.19**	**4**	**9**	
**CHEMBL2440387**	C25H23ClN4O4S	3.38	4	10	4.94
**CHEMBL2424928**	C24H26FN5O4	3.51	4	10	
**CHEMBL1916543**	C23H28N6O4	3.69	4	10	
**CHEMBL2041188**	C32H30N6O4	4.64	4	10	
**CHEMBL3400819**	C26H26FN5O4	4.91	4	10	
**CHEMBL256060**	C26H26ClN5O4	4.96	4	10	
**CHEMBL513921**	C23H34N6O4	5.24	4	10	
**CHEMBL3604800**	C28H32FN5O4	5.52	4	10	
**CHEMBL3605145**	C29H32FN5O4	6.30	4	10	
**CHEMBL3605140**	C27H30FN5O4	6.40	4	10	
**CHEMBL3985847**	C21H26N6O4S	2.40	4	11	4.60
**CHEMBL1916544**	C22H27N7O4	4.09	4	11	
**CHEMBL1091605**	C29H36F3N3O4S	4.52	4	11	
**CHEMBL2069410**	C23H19F4N3O4	4.60	4	11	
**CHEMBL192**	C22H30N6O4S	5.48	4	11	
**CHEMBL3422758**	C27H29F2N5O4	5.76	4	11	
**CHEMBL3422952**	C26H27F2N5O4	9.14	4	11	
**CHEMBL3799831**	C25H26FN3O5	4.03	5	9	5.00
**CHEMBL3612928**	C24H28N4O5	4.70	5	9	
**CHEMBL3786311**	C26H30N4O5	4.80	5	9	
**CHEMBL1091664**	C28H25ClN2O5S	5.00	5	9	
**CHEMBL3612926**	C25H30N4O5	5.11	5	9	
**CHEMBL429458**	C25H30FN3O5	5.20	5	9	
**CHEMBL387178**	C23H20F2N2O5	8.21	5	9	
**CHEMBL3921669**	C23H26N4O5S	2.22	5	10	4.99
**CHEMBL605785**	C27H27FN4O5	4.02	5	10	
**CHEMBL247690**	C29H33F2N3O5	4.47	5	10	
**CHEMBL245642**	C25H25F2N3O5	5.50	5	10	
**CHEMBL217707**	C23H19F3N2O5	8.25	5	10	
**CHEMBL473**	C19H27N3O5S2	8.39	5	10	

Table S6. Investigation of the influence of nitrogen on the pIC_{50} activity.

Name	Molecular formula	pIC50	N	Hetatoms	median value
**CHEMBL3314420**	C15H13ClFN	4.30	1	3	5.69
**CHEMBL1684047**	C16H23Cl2N	4.40	1	3	
**CHEMBL2325209**	$C26H28NO2^{+}$	5.54	1	3	
**CHEMBL299390**	C19H19ClFN	5.83	1	3	
**CHEMBL2324248**	$C20H20NO2^{+}$	6.12	1	3	
**CHEMBL607**	C15H21NO2	6.49	1	3	
**CHEMBL70**	C17H19NO3	3.00	1	4	4.89
**CHEMBL1671896**	C11H14F3N	3.27	1	4	
**CHEMBL3220615**	C19H21NO3	4.89	1	4	
**CHEMBL53866**	C17H15ClFNO	5.66	1	4	
**CHEMBL96153**	C24H31NO3	7.52	1	4	
**CHEMBL2324239**	$C24H18Cl2NO2^{+}$	4.51	1	5	6.10
**CHEMBL914**	C32H39NO4	4.67	1	5	
**CHEMBL378547**	C21H21F2NO2	5.11	1	5	
**CHEMBL196983**	C21H27NO4	6.10	1	5	
**CHEMBL2324243**	$C26H32NO4^{+}$	6.80	1	5	
**CHEMBL2324245**	$C24H26Cl2NO2^{+}$	7.22	1	5	
**CHEMBL572163**	C26H31NO4	7.42	1	5	
**CHEMBL284348**	C5H6N2	2.36	2	2	5.06
**CHEMBL3**	C10H14N2	3.61	2	2	
**CHEMBL1086033**	C17H22N2	3.67	2	2	
**CHEMBL87045**	C18H18N2	5.06	2	2	
**CHEMBL459176**	C20H22N2	5.16	2	2	
**CHEMBL11**	C19H24N2	5.47	2	2	
**CHEMBL1671894**	C27H26N2	7.75	2	2	
**CHEMBL1087**	C15H22N2O	3.81	2	3	5.16
**CHEMBL1083707**	C20H24N2O	4.67	2	3	
**CHEMBL513258**	C19H19FN2	5.05	2	3	
**CHEMBL1945694**	C16H15ClN2	5.16	2	3	

Table S6 (Cont.d)

**CHEMBL191054**	**C13H17ClN2**	**5.60**	**2**	**3**	
**CHEMBL505**	C16H19ClN2	5.96	2	3	
**CHEMBL1090528**	C19H24N2S	6.21	2	3	
**CHEMBL16**	C15H12N2O2	4.00	2	4	5.92
**CHEMBL3356251**	C24H36N2O2	4.01	2	4	
**CHEMBL3786542**	C26H26N2O2	4.21	2	4	
**CHEMBL3356249**	C25H38N2O2	4.30	2	4	
**CHEMBL3314426**	C15H14F2N2	4.66	2	4	
**CHEMBL1086273**	C19H23FN2O	4.68	2	4	
**CHEMBL2333646**	C19H23BrN2O	4.89	2	4	
**CHEMBL3185736**	C22H20N2O2	4.91	2	4	
**CHEMBL4162513**	C35H34N2O2	5.10	2	4	
**CHEMBL4450665**	C23H28N2O2	5.92	2	4	
**CHEMBL3323181**	C22H24N2OS	6.00	2	4	
**CHEMBL1092377**	C19H21FN2S	6.48	2	4	
**CHEMBL1346**	C28H30N2O2	7.10	2	4	
**CHEMBL2046892**	C22H22F2N2	7.40	2	4	
**CHEMBL1223951**	C21H24N2O2	9.21	2	4	
**CHEMBL1257938**	C22H26N2O2	9.21	2	4	
**CHEMBL1257937**	C22H26N2O2	9.28	2	4	
**CHEMBL1257820**	C23H28N2O2	9.60	2	4	
**CHEMBL1257821**	C22H26N2O2	9.85	2	4	
**CHEMBL4640366**	C33H34N2O3	4.02	2	5	6.17
**CHEMBL4209354**	C15H20N2O2S	4.07	2	5	
**CHEMBL3787069**	C26H26N2O3	4.35	2	5	
**CHEMBL1080790**	C16H22Cl2N2O	4.78	2	5	
**CHEMBL1080969**	C16H22Cl2N2O	5.11	2	5	
**CHEMBL498572**	C19H22N2O3	5.23	2	5	
**CHEMBL4100591**	C27H34F2N2O	5.29	2	5	
**CHEMBL1224697**	C22H28N2O2S	5.90	2	5	

Table S6 (Cont.d)

**CHEMBL376488**	C32H31BrN2O2	**6.43**	**2**	**5**	
**CHEMBL1671893**	C23H30N2O3	6.60	2	5	
**CHEMBL998**	C22H23ClN2O2	6.77	2	5	
**CHEMBL195180**	C24H27ClN2OS	7.52	2	5	
**CHEMBL1642487**	C28H28N2O3	9.09	2	5	
**CHEMBL1642479**	C28H28N2O3	9.17	2	5	
**CHEMBL1257577**	C19H22N2O2S	9.37	2	5	
**CHEMBL1257578**	C20H24N2O2S	9.59	2	5	
**CHEMBL270190**	C25H32N2O4	3.10	2	6	5.90
**CHEMBL519266**	C22H28N2O4	3.11	2	6	
**CHEMBL1000**	C21H25ClN2O3	4.52	2	6	
**CHEMBL1938433**	C23H24N2O4	5.42	2	6	
**CHEMBL3219616**	C24H30N2O3S	5.80	2	6	
**CHEMBL1224699**	C21H26N2O3S	5.90	2	6	
**CHEMBL561279**	C25H34N2O3S	5.93	2	6	
**CHEMBL723**	C24H26N2O4	6.46	2	6	
**CHEMBL4173253**	C26H34N2O3S	7.10	2	6	
**CHEMBL533**	C20H36N2O3S	8.00	2	6	
**CHEMBL1258280**	C22H24N2O4	9.21	2	6	
**CHEMBL374731**	C10H14N2O5	2.30	2	7	5.05
**CHEMBL3329814**	C21H28N2O5	4.09	2	7	
**CHEMBL2158823**	C24H28Cl2N2O3	4.50	2	7	
**CHEMBL2333636**	C24H28F4N2O	4.75	2	7	
**CHEMBL2333616**	C20H22ClF3N2O	4.92	2	7	
**CHEMBL2333607**	C25H30F4N2O	5.05	2	7	
**CHEMBL2333615**	C23H26F4N2O	5.05	2	7	
**CHEMBL2158815**	C24H28Cl2N2O3	5.30	2	7	
**CHEMBL246634**	C21H23FN2O4	5.58	2	7	
**CHEMBL1098847**	C24H21Cl3N2O2	5.58	2	7	
**CHEMBL2387265**	C31H29FN2O4	6.09	2	7	
**CHEMBL384487**	C24H23FN2O4	7.69	2	7	

Table S6 (Cont.d)

**CHEMBL217593**	C23H23FN2O4	8.27	2	7	
**CHEMBL193**	C17H18N2O6	4.30	2	8	5.41
**CHEMBL2382343**	C29H32F2N2O4	4.32	2	8	
**CHEMBL2440407**	C23H26N2O5S	5.02	2	8	
**CHEMBL2387229**	C26H26ClFN2O4	5.80	2	8	
**CHEMBL214021**	C23H21FN2O5	7.82	2	8	
**CHEMBL217789**	C23H21ClN2O5	8.44	2	8	
**CHEMBL216323**	C23H19FN2O6	4.07	2	9	5.00
**CHEMBL4092041**	C29H31F3N2O4	4.07	2	9	
**CHEMBL2158050**	C25H29FN2O4S2	4.63	2	9	
**CHEMBL1091664**	C28H25ClN2O5S	5.00	2	9	
**CHEMBL557306**	C21H24ClF3N2O3	5.99	2	9	
**CHEMBL217442**	C24H23FN2O6	7.80	2	9	
**CHEMBL387178**	C23H20F2N2O5	8.21	2	9	
**CHEMBL428594**	C13H21N3O	3.86	3	4	5.40
**CHEMBL217707**	C17H25N3O	3.94	3	4	
**CHEMBL213715**	C17H33N3O	4.17	3	4	
**CHEMBL1091218**	C20H31N3O	4.42	3	4	
**CHEMBL1097456**	C22H23N3O	4.70	3	4	
**CHEMBL3417745**	C21H29N3O	4.92	3	4	
**CHEMBL402624**	C22H33N3O	5.40	3	4	
**CHEMBL490026**	C21H22ClN3	5.44	3	4	
**CHEMBL497171**	C20H25N3O	5.47	3	4	
**CHEMBL457930**	C20H23N3O	5.68	3	4	
**CHEMBL299499**	C17H21N3S	5.70	3	4	
**CHEMBL640**	C22H26ClN3	5.96	3	4	
**CHEMBL219803**	C23H25N3O	6.13	3	4	
**CHEMBL219128**	C15H23N3O2	4.00	3	5	5.18
**CHEMBL219074**	C19H25N3O2	4.12	3	5	
**CHEMBL1089159**	C23H27N3O2	4.14	3	5	
**CHEMBL517**	C22H23N3O2	4.27	3	5	

Table S6 (Cont.d)

CHEMBL1094041	C29H29N3O2	4.55	3	5	
**CHEMBL2441636**	C22H33N3O2	4.72	3	5	
**CHEMBL2177304**	C27H24FN3O	5.07	3	5	
**CHEMBL487063**	C23H27N3O2	5.10	3	5	
**CHEMBL485242**	C25H29N3O2	5.14	3	5	
**CHEMBL478615**	C23H27N3O2	5.17	3	5	
**CHEMBL2326478**	C18H18FN3S	5.17	3	5	
**CHEMBL476579**	C24H29N3O2	5.18	3	5	
**CHEMBL479242**	C23H27N3O2	5.18	3	5	
**CHEMBL3121096**	C20H25N3O2	5.22	3	5	
**CHEMBL1819137**	C22H25N3O2	5.23	3	5	
**CHEMBL488249**	C24H27N3O2	5.25	3	5	
**CHEMBL384400**	C27H36ClN3O	5.48	3	5	
**CHEMBL487064**	C23H27N3O2	5.55	3	5	
**CHEMBL478616**	C23H27N3O2	5.80	3	5	
**CHEMBL562833**	C22H30ClN3O	6.10	3	5	
**CHEMBL259732**	C20H27N3O2	9.19	3	5	
**CHEMBL410832**	C23H25N3O2	9.39	3	5	
**CHEMBL4787638**	C21H18ClN3O2	2.08	3	6	6.20
**CHEMBL1823042**	C20H28FN3O2	5.00	3	6	
**CHEMBL4089699**	C19H19N3O2S	5.42	3	6	
**CHEMBL563926**	C21H26ClN3O2	6.20	3	6	
**CHEMBL1108**	C22H22FN3O2	7.49	3	6	
**CHEMBL1423**	C28H29F2N3O	8.52	3	6	
**CHEMBL1258503**	C22H25N3O3	9.14	3	6	
**CHEMBL8**	C17H18FN3O3	3.02	3	7	5.04
**CHEMBL4855895**	C21H24FN3O3	3.53	3	7	
**CHEMBL4847738**	C20H23N3O4	4.05	3	7	
**CHEMBL583**	C19H22FN3O3	4.30	3	7	
**CHEMBL218836**	C18H24F3N3O	4.40	3	7	
**CHEMBL2314058**	C25H34F3N3O	4.83	3	7	

Table S6 (Cont.d)

**CHEMBL3973288**	**C20H22FN3O2S**	**4.84**	**3**	**7**	
**CHEMBL3115194**	C26H32BrN3O3	4.90	3	7	
**CHEMBL1957792**	C27H30ClN3O3	4.99	3	7	
**CHEMBL3703271**	C22H23N3O4	5.08	3	7	
**CHEMBL246815**	C22H26FN3O3	5.13	3	7	
**CHEMBL398478**	C25H28FN3O3	5.21	3	7	
**CHEMBL250533**	C25H28ClN3O3	5.48	3	7	
**CHEMBL3086038**	C30H33BrClN3O2	5.82	3	7	
**CHEMBL3806193**	C19H23Cl2N3O2	5.84	3	7	
**CHEMBL17423**	C22H25N3O4	5.96	3	7	
**CHEMBL3806153**	C18H21ClFN3O2	6.01	3	7	
**CHEMBL45816**	C29H38FN3O3	6.24	3	7	
**CHEMBL33**	C18H20FN3O4	3.04	3	8	4.57
**CHEMBL31**	C19H22FN3O4	3.89	3	8	
**CHEMBL32**	C21H24FN3O4	4.10	3	8	
**CHEMBL2333619**	C22H27F4N3O	4.10	3	8	
**CHEMBL2314064**	C24H32F3N3O2	4.41	3	8	
**CHEMBL540634**	C26H31Cl2N3O3	4.50	3	8	
**CHEMBL3577935**	C23H34F3N3O2	4.50	3	8	
**CHEMBL2164565**	C19H18F3N3O2	4.63	3	8	
**CHEMBL3964723**	C23H27Cl2N3O3	5.00	3	8	
**CHEMBL2158771**	C27H31Cl2N3O3	5.30	3	8	
**CHEMBL2181489**	C23H20F3N3O2	5.52	3	8	
**CHEMBL2441431**	C22H23N3O5	5.89	3	8	
**CHEMBL453894**	C25H26F3N3O2	6.46	3	8	
**CHEMBL568571**	C27H26F3N3O2	6.85	3	8	
**CHEMBL3799831**	C25H26FN3O5	4.03	3	9	5.14
**CHEMBL248296**	C27H31F2N3O4	4.19	3	9	
**CHEMBL4084170**	C15H18F5N3S	4.69	3	9	
**CHEMBL3422244**	C18H18F3N3O2S	4.71	3	9	
**CHEMBL1083118**	C17H19F2N3O3S	4.89	3	9	

Table S6 (Cont.d)

**CHEMBL2164393**	**C26H24F3N3O3**	**5.14**	**3**	**9**	
**CHEMBL429458**	C25H30FN3O5	5.20	3	9	
**CHEMBL4170966**	C24H21Cl2N3O4	5.33	3	9	
**CHEMBL2164389**	C24H20F3N3O3	5.34	3	9	
**CHEMBL549635**	C29H33F4N3O2	6.20	3	9	
**CHEMBL1729**	C23H29ClFN3O4	8.19	3	9	
**CHEMBL247690**	C29H33F2N3O5	4.47	3	10	5.50
**CHEMBL2164047**	C24H19F4N3O3	5.28	3	10	
**CHEMBL245642**	C25H25F2N3O5	5.50	3	10	
**CHEMBL4081080**	C25H17F6N3O	7.17	3	10	
**CHEMBL473**	C19H27N3O5S2	8.39	3	10	
**CHEMBL1091605**	C25H34N4O	4.62	4	5	5.49
**CHEMBL2069410**	C18H22N4O	4.68	4	5	
**CHEMBL2164375**	C20H24N4O	4.71	4	5	
**CHEMBL4076061**	C16H18N4S	4.90	4	5	
**CHEMBL2207664**	C20H22N4O	4.94	4	5	
**CHEMBL435882**	C15H14N4O	5.01	4	5	
**CHEMBL4213196**	C21H28N4S	5.33	4	5	
**CHEMBL217133**	C17H18N4O	5.64	4	5	
**CHEMBL429115**	C31H38N4O	6.24	4	5	
**CHEMBL1125**	C26H28N4O	6.28	4	5	
**CHEMBL4096683**	C17H26N4O	6.63	4	5	
**CHEMBL565829**	C18H19ClN4	6.72	4	5	
**CHEMBL1956112**	C17H20N4S	6.74	4	5	
**CHEMBL1110**	C19H21FN4	7.56	4	5	
**CHEMBL58387**	C18H19ClN4O	3.85	4	6	5.61
**CHEMBL3775729**	C26H35FN4O	4.88	4	6	
**CHEMBL1087493**	C21H34N4S2	4.88	4	6	
**CHEMBL42**	C20H21FN4O	5.11	4	6	
**CHEMBL715**	C20H22N4OS	5.23	4	6	
**CHEMBL61301**	C24H26N4OS	5.29	4	6	

Table S6 (Cont.d)

**CHEMBL1823047**	**C22H30N4O2**	**5.54**	**4**	**6**	
**CHEMBL4065208**	C23H22N4O2	5.56	4	6	
**CHEMBL2146805**	C20H21ClN4O	5.60	4	6	
**CHEMBL4104102**	C20H22N4OS	5.61	4	6	
**CHEMBL2177905**	C24H16F2N4	5.80	4	6	
**CHEMBL1083463**	C26H26Cl2N4	6.22	4	6	
**CHEMBL3774817**	C25H25FN4O	6.59	4	6	
**CHEMBL522456**	CHEMBL522456	7.00	4	6	
**CHEMBL3775282**	C26H28N4O2	7.16	4	6	
**CHEMBL12780**	C24H27FN4O	8.16	4	6	
**CHEMBL2146810**	C23H27ClN4O	8.40	4	6	
**CHEMBL296419**	C28H31FN4O	9.05	4	6	
**CHEMBL22**	C14H18N4O3	3.62	4	7	5.44
**CHEMBL3582294**	C25H18F2N4O	4.03	4	7	
**CHEMBL388978**	C28H26N4O3	4.04	4	7	
**CHEMBL4781551**	C24H21ClN4O2	4.08	4	7	
**CHEMBL1092106**	C20H24ClFN4O	5.04	4	7	
**CHEMBL1091834**	C25H26ClFN4O	5.42	4	7	
**CHEMBL1939739**	C32H35FN4O2	5.44	4	7	
**CHEMBL3764774**	C28H28N4O3	5.70	4	7	
**CHEMBL3775050**	C25H24F2N4O	6.20	4	7	
**CHEMBL1084400**	C20H21F3N4	6.80	4	7	
**CHEMBL708**	C21H21ClN4OS	6.92	4	7	
**CHEMBL2146854**	C23H26N4O3	7.33	4	7	
**CHEMBL12713**	C24H26ClFN4O	8.57	4	7	
**CHEMBL520463**	C28H24N4O4	4.05	4	8	5.01
**CHEMBL485123**	C20H23F3N4O	4.07	4	8	
**CHEMBL3787345**	C25H28N4O4	4.51	4	8	
**CHEMBL602875**	C26H34N4O4	4.72	4	8	
**CHEMBL2178221**	C28H29ClN4O3	4.82	4	8	
**CHEMBL3612814**	C24H28N4O4	4.85	4	8	

Table S6 (Cont.d)

**CHEMBL1084617**	**C24H28N4O4**	**4.96**	**4**	**8**	
**CHEMBL3786346**	C25H26N4O4	5.05	4	8	
**CHEMBL207220**	C24H40N4O3S	5.40	4	8	
**CHEMBL2207738**	C23H26N4O3S	5.60	4	8	
**CHEMBL3703237**	C19H17ClN4O3	5.66	4	8	
**CHEMBL1956991**	C23H26ClFN4O2	5.89	4	8	
**CHEMBL1621**	C23H27FN4O3	6.00	4	8	
**CHEMBL128007**	C28H28Cl2N4O2	7.00	4	8	
**CHEMBL3612928**	C24H28N4O5	4.70	4	9	5.11
**CHEMBL3786311**	C26H30N4O5	4.80	4	9	
**CHEMBL596700**	C20H23F3N4O2	4.88	4	9	
**CHEMBL1095476**	C28H25Cl3N4O2	5.06	4	9	
**CHEMBL589833**	C24H25Cl2FN4O2	5.10	4	9	
**CHEMBL3612926**	C25H30N4O5	5.11	4	9	
**CHEMBL2170611**	C17H21F3N4O2	5.16	4	9	
**CHEMBL4243499**	C19H16F3N5O	5.25	4	9	
**CHEMBL244083**	C29H36N4O4S	5.60	4	9	
**CHEMBL550410**	C25H26F4N4O	5.92	4	9	
**CHEMBL3983509**	C20H20ClFN4O2S	6.70	4	9	
**CHEMBL3921669**	C23H26N4O5S	2.22	4	10	5.04
**CHEMBL2440387**	C25H23ClN4O4S	3.38	4	10	
**CHEMBL605785**	C27H27FN4O5	4.02	4	10	
**CHEMBL2164365**	C23H19F3N4O3	5.04	4	10	
**CHEMBL560063**	C24H23Cl2F3N4O	5.46	4	10	
**CHEMBL553196**	C32H32F4N4O2	5.76	4	10	
**CHEMBL375330**	C34H38ClF3N4O2	6.77	4	10	
**CHEMBL1782574**	C23H35F3N4O3S	2.41	4	11	4.81
**CHEMBL2204260**	C22H25F3N4O3S	4.62	4	11	
**CHEMBL2204270**	C24H26F4N4O3	5.00	4	11	
**CHEMBL2147223**	C22H20ClF3N4O3	5.40	4	11	
**CHEMBL256154**	C27H32F6N4O	5.85	4	11	

Table S6 (Cont.d)

**CHEMBL491571**	**C15H24N4O6S2**	**2.10**	**4**	**12**	**4.92**
**CHEMBL2147316**	C22H19F5N4O3	4.60	4	12	
**CHEMBL2147303**	C22H19F5N4O3	4.70	4	12	
**CHEMBL3764895**	C29H31Cl2F3N4O3	5.13	4	12	
**CHEMBL3764357**	C29H31Cl2F3N4O3	5.20	4	12	
**CHEMBL3765012**	C30H31Cl2F3N4O3	5.51	4	12	
**CHEMBL370072**	C24H31N5O	4.64	5	6	5.91
**CHEMBL401608**	C21H24ClN5	5.05	5	6	
**CHEMBL487708**	C23H16FN5	5.51	5	6	
**CHEMBL237191**	C25H19N5O	6.30	5	6	
**CHEMBL4212656**	C22H27N5O	6.52	5	6	
**CHEMBL494406**	C32H33N5O	7.62	5	6	
**CHEMBL2177904**	C21H25N5OS	3.72	5	7	5.26
**CHEMBL4081790**	C22H22ClN5O	4.22	5	7	
**CHEMBL4096145**	C21H21N5OS	4.52	5	7	
**CHEMBL329067**	C26H29N5O2	4.60	5	7	
**CHEMBL578834**	C21H23ClFN5	4.64	5	7	
**CHEMBL4127458**	C17H22FN5O	4.80	5	7	
**CHEMBL2069925**	C22H23N5O2	4.89	5	7	
**CHEMBL1957012**	C28H33N5O2	4.96	5	7	
**CHEMBL512975**	C24H23N5O2	5.08	5	7	
**CHEMBL2180842**	C21H15F2N5	5.16	5	7	
**CHEMBL523374**	C27H23N5O2	5.36	5	7	
**CHEMBL3218884**	C24H29N5O2	5.40	5	7	
**CHEMBL498042**	C25H22FN5O	5.70	5	7	
**CHEMBL523821**	C23H31N5OS	5.90	5	7	
**CHEMBL497048**	C19H20ClN5O	5.90	5	7	
**CHEMBL2336331**	C25H27N5O2	5.99	5	7	
**CHEMBL2151218**	C28H31N5OS	6.20	5	7	
**CHEMBL243901**	C29H25N5O2	6.50	5	7	
**CHEMBL2146812**	C23H24FN5S	7.00	5	7	

Table S6 (Cont.d)

**CHEMBL526466**	**C25H23N5O2**	**7.10**	**5**	**7**	
**CHEMBL3804950**	C20H21F2N5O	4.01	5	8	5.70
**CHEMBL3216126**	C25H35Cl2N5O	4.65	5	8	
**CHEMBL1939742**	C30H37N5O2S	4.75	5	8	
**CHEMBL1093696**	C24H25ClFN5O	4.92	5	8	
**CHEMBL465417**	C25H23N5O3	5.04	5	8	
**CHEMBL506163**	C22H19ClFN5O	5.20	5	8	
**CHEMBL3218650**	C27H30ClN5O2	5.60	5	8	
**CHEMBL1080489**	C22H27N5O2S	5.80	5	8	
**CHEMBL3318989**	C23H24ClN5O2	6.00	5	8	
**CHEMBL244280**	C26H28FN5OS	6.60	5	8	
**CHEMBL2146813**	C38H48ClN5O2	6.80	5	8	
**CHEMBL525413**	C29H24ClN5O2	7.09	5	8	
**CHEMBL244278**	C26H28ClN5OS	7.10	5	8	
**CHEMBL2146853**	C20H23N5O3	7.60	5	8	
**CHEMBL2165068**	C25H26FN5O3	4.24	5	9	5.42
**CHEMBL3650850**	C21H23N5O4	4.68	5	9	
**CHEMBL255968**	C25H24ClN5O3	5.00	5	9	
**CHEMBL4075071**	C19H16F3N5O	5.25	5	9	
**CHEMBL255645**	C28H31Cl2N5O2	5.36	5	9	
**CHEMBL1927161**	C24H29N5O4	5.40	5	9	
**CHEMBL2158625**	C20H19Cl2N5OS	5.44	5	9	
**CHEMBL489324**	C28H42ClF2N5O	5.68	5	9	
**CHEMBL4213486**	C19H15F2N5O2	5.70	5	9	
**CHEMBL2158629**	C20H21Cl2N5OS	5.85	5	9	
**CHEMBL2177305**	C28H23ClFN5O2	5.89	5	9	
**CHEMBL1081747**	C21H23ClFN5OS	6.40	5	9	
**CHEMBL2424928**	C24H26FN5O4	3.51	5	10	5.71
**CHEMBL2315921**	C26H28F3N5O2	4.77	5	10	
**CHEMBL3400819**	C26H26FN5O4	4.91	5	10	

Table S6 (Cont.d)

**CHEMBL256060**	**C26H26ClN5O4**	**4.96**	**5**	**10**	
**CHEMBL551281**	C26H34F3N5O2	5.52	5	10	
**CHEMBL3604800**	C28H32FN5O4	5.52	5	10	
**CHEMBL2403108**	C28H36ClN5O3S	5.90	5	10	
**CHEMBL537847**	C23H29Cl2N5O3	6.25	5	10	
**CHEMBL3605145**	C29H32FN5O4	6.30	5	10	
**CHEMBL3605140**	C27H30FN5O4	6.40	5	10	
**CHEMBL1081746**	C23H26F3N5S2	6.46	5	10	
**CHEMBL401576**	C25H26F3N5OS	6.90	5	10	
**CHEMBL3400817**	C26H26FN5O5	3.76	5	11	5.76
**CHEMBL3746204**	C22H17F4N5O2	5.24	5	11	
**CHEMBL2331648**	C20H22F3N5O3	5.34	5	11	
**CHEMBL4075908**	C20H21BrFN5O3S	5.46	5	11	
**CHEMBL3422758**	C27H29F2N5O4	5.76	5	11	
**CHEMBL1082111**	C22H23F4N5OS	5.90	5	11	
**CHEMBL3605123**	C30H36FN5O5	6.16	5	11	
**CHEMBL3422952**	C26H27F2N5O4	9.14	5	11	
**CHEMBL3422978**	C26H26F3N5O3	9.41	5	11	
**CHEMBL3425799**	C26H25F2N5O5	3.48	5	12	4.08
**CHEMBL1784523**	C36H40F3N5O3S	4.00	5	12	
**CHEMBL451887**	C40H57N5O7	4.04	5	12	
**CHEMBL3342693**	C23H22F5N5O2	4.08	5	12	
**CHEMBL3605131**	C29H32FN5O6	4.91	5	12	
**CHEMBL550535**	C27H33F6N5O	5.41	5	12	
**CHEMBL3605006**	C31H34FN5O6	5.80	5	12	
**CHEMBL3422957**	C14H16N6O	4.03	6	7	5.28
**CHEMBL231396**	C21H26N6S	4.71	6	7	
**CHEMBL2147024**	C23H26N6O	5.09	6	7	
**CHEMBL4224807**	C23H19FN6	5.28	6	7	
**CHEMBL575241**	C20H20N6O	5.42	6	7	

Table S6 (Cont.d)

**CHEMBL2147021**	**C20H15FN6**	**5.57**	**6**	**7**	
**CHEMBL4279819**	C16H18N6O	8.07	6	7	
**CHEMBL399726**	C20H24N6O2	3.78	6	8	5.60
**CHEMBL4858551**	C19H19FN6O	4.01	6	8	
**CHEMBL256653**	C23H26N6OS	4.30	6	8	
**CHEMBL257901**	C28H30N6OS	4.90	6	8	
**CHEMBL270239**	C30H34N6OS	4.90	6	8	
**CHEMBL515001**	C21H25FN6O	4.96	6	8	
**CHEMBL2325997**	C20H23ClN6O	5.14	6	8	
**CHEMBL3287218**	C29H32N6OS	5.16	6	8	
**CHEMBL270852**	C32H38N6OS	5.40	6	8	
**CHEMBL271909**	C28H30N6S2	5.50	6	8	
**CHEMBL272086**	C32H38N6OS	5.60	6	8	
**CHEMBL2178521**	C24H21FN6O	5.75	6	8	
**CHEMBL3415593**	C19H26N6O2	5.77	6	8	
**CHEMBL478462**	C25H38N6O2	5.84	6	8	
**CHEMBL515025**	C24H36N6O2	6.15	6	8	
**CHEMBL478011**	C25H38N6O2	6.38	6	8	
**CHEMBL408169**	C28H32N6OS	6.40	6	8	
**CHEMBL94454**	C24H25FN6O	6.46	6	8	
**CHEMBL233047**	C20H32Cl2N6	6.52	6	8	
**CHEMBL256442**	C29H32N6S2	6.80	6	8	
**CHEMBL397429**	C28H30N6OS	7.30	6	8	
**CHEMBL2324519**	C20H21FN6O2	3.67	6	9	4.89
**CHEMBL3290351**	C24H27FN6O2	4.05	6	9	
**CHEMBL3290344**	C24H26N6O3	4.13	6	9	
**CHEMBL3608687**	C16H15ClN6OS	4.18	6	9	
**CHEMBL4094682**	C22H27ClN6O2	4.34	6	9	
**CHEMBL3218891**	C27H32N6O3	4.37	6	9	
**CHEMBL2441417**	C24H26N6O3	4.89	6	9	

Table S6 (Cont.d)

**CHEMBL502288**	**C23H22N6O2S**	**5.11**	**6**	**9**	
**CHEMBL402015**	C27H27BrN6OS	5.20	6	9	
**CHEMBL514042**	C25H38N6O3	6.05	6	9	
**CHEMBL498411**	C22H19Cl3N6	6.46	6	9	
**CHEMBL3422973**	C27H28N6O3	8.68	6	9	
**CHEMBL3422970**	C27H28N6O3	9.06	6	9	
**CHEMBL1916543**	C23H28N6O4	3.69	6	10	4.66
**CHEMBL4112037**	C16H14F2N6OS	4.16	6	10	
**CHEMBL3221500**	C22H21ClN6O3	4.20	6	10	
**CHEMBL3794265**	C23H25FN6O3	4.38	6	10	
**CHEMBL3221488**	C23H23ClN6O3	4.50	6	10	
**CHEMBL3425929**	C28H33FN6O3	4.51	6	10	
**CHEMBL2041188**	C32H30N6O4	4.64	6	10	
**CHEMBL2324520**	C20H20F2N6O2	4.68	6	10	
**CHEMBL563791**	C28H29F3N6O	4.90	6	10	
**CHEMBL513921**	C23H34N6O4	5.24	6	10	
**CHEMBL3408394**	C37H24N6O2S2	5.24	6	10	
**CHEMBL562285**	C24H27N3O2	5.27	6	10	
**CHEMBL1081867**	C22H23F3N6S	6.33	6	10	
**CHEMBL390649**	C28H31F3N6S	7.50	6	10	
**CHEMBL3985847**	C21H26N6O4S	2.40	6	11	4.70
**CHEMBL3964789**	C21H22F4N6O	3.17	6	11	
**CHEMBL2165057**	C25H30N6O5	4.06	6	11	
**CHEMBL3703025**	C22H25F3N6O2	4.50	6	11	
**CHEMBL3407784**	C20H19ClN6O3S	4.89	6	11	
**CHEMBL3318984**	C23H23F3N6O2	5.00	6	11	
**CHEMBL192**	C22H30N6O4S	5.48	6	11	
**CHEMBL402016**	C28H27F3N6OS	6.00	6	11	
**CHEMBL254316**	C20H21FN6O5	2.50	6	12	4.60
**CHEMBL3608744**	C28H31ClN6O4S	4.09	6	12	

Table S6 (Cont.d)

**CHEMBL3425807**	**C25H25FN6O5**	**4.52**	**6**	**12**	
**CHEMBL2206791**	C23H21F3N6O3	4.58	6	12	
**CHEMBL2336323**	C26H30F2N6O4	4.62	6	12	
**CHEMBL2064657**	C28H23ClF2N6O3	4.91	6	12	
**CHEMBL3288030**	C44H53FN6O5	5.20	6	12	
**CHEMBL2441412**	C24H23F3N6O3	5.29	6	12	
**CHEMBL195378**	C22H21N7O2	4.30	7	9	5.60
**CHEMBL474484**	C26H34ClN7O	4.53	7	9	
**CHEMBL2147022**	C21H22ClN7O	4.70	7	9	
**CHEMBL2180070**	C25H31N7OS	5.60	7	9	
**CHEMBL3593771**	C23H31N7O2	5.73	7	9	
**CHEMBL262341**	C28H25N7O2	5.85	7	9	
**CHEMBL399525**	C34H31N7O2	5.91	7	9	
**CHEMBL484816**	C22H27N7O4	4.09	7	11	4.90
**CHEMBL2325729**	C23H22ClN7O3	4.40	7	11	
**CHEMBL3262358**	C23H20ClN7O3	4.90	7	11	
**CHEMBL245119**	C31H27N7O3S	5.82	7	11	
**CHEMBL429761**	C28H28F3N7S	6.60	7	11	

Table S7. Investigation of the influence of Sulfur on the pIC_{50} activity

Name	Molecular formula	pIC50	S	Heteroatoms	median
**CHEMBL284348**	C5H6N2	2.36	0	2	5.16
**CHEMBL3**	C10H14N2	3.61	0	2	
**CHEMBL1086033**	C17H22N2	3.67	0	2	
**CHEMBL87045**	C18H18N2	5.06	0	2	
**CHEMBL459176**	C20H22N2	5.16	0	2	
**CHEMBL1684059**	C20H28ClN	5.37	0	2	

Table S7 (cont.d)

**CHEMBL11**	C19H24N2	5.47	0	2	
**CHEMBL83**	C26H29NO	6.01	0	2	
**CHEMBL1671894**	C27H26N2	7.75	0	2	
**CHEMBL1087**	C15H22N2O	3.81	0	3	5.60
**CHEMBL3314420**	C15H13ClFN	4.30	0	3	
**CHEMBL1684047**	C16H23Cl2N	4.40	0	3	
**CHEMBL1083707**	C20H24N2O	4.67	0	3	
**CHEMBL513258**	C19H19FN2	5.05	0	3	
**CHEMBL1945694**	C16H15ClN2	5.16	0	3	
**CHEMBL2325209**	$C26H28NO2^{+}$	5.54	0	3	
**CHEMBL191054**	C13H17ClN2	5.60	0	3	
**CHEMBL497171**	C15H17N3	5.80	0	3	
**CHEMBL299390**	C19H19ClFN	5.83	0	3	
**CHEMBL457930**	C20H19N3	5.85	0	3	
**CHEMBL505**	C16H19ClN2	5.96	0	3	
**CHEMBL2324248**	$C20H20NO2^{+}$	6.12	0	3	
**CHEMBL299499**	C17H22IN3	6.31	0	3	
**CHEMBL607**	C15H21NO2	6.49	0	3	
**CHEMBL70**	C17H19NO3	3.00	0	4	5.01
**CHEMBL1671896**	C11H14F3N	3.27	0	4	
**CHEMBL640**	C13H21N3O	3.86	0	4	
**CHEMBL219803**	C17H25N3O	3.94	0	4	
**CHEMBL16**	C15H12N2O2	4.00	0	4	
**CHEMBL3356251**	C24H36N2O2	4.01	0	4	
**CHEMBL219128**	C17H33N3O	4.17	0	4	
**CHEMBL3786542**	C26H26N2O2	4.21	0	4	
**CHEMBL3356249**	C25H38N2O2	4.30	0	4	
**CHEMBL219074**	C20H31N3O	4.42	0	4	
**CHEMBL3314426**	C15H14F2N2	4.66	0	4	

Table S7 (Cont.d)

**CHEMBL1086273**	**C19H23FN2O**	**4.68**	**0**	**4**	
**CHEMBL1089159**	C22H23N3O	4.70	0	4	
**CHEMBL2333646**	C19H23BrN2O	4.89	0	4	
**CHEMBL3220615**	C19H21NO3	4.89	0	4	
**CHEMBL3185736**	C22H20N2O2	4.91	0	4	
**CHEMBL517**	C21H29N3O	4.92	0	4	
**CHEMBL4162513**	C35H34N2O2	5.10	0	4	
**CHEMBL562948**	C22H33N3O	5.40	0	4	
**CHEMBL1083407**	C21H22ClN3	5.44	0	4	
**CHEMBL1090175**	C20H25N3O	5.47	0	4	
**CHEMBL53866**	C17H15ClFNO	5.66	0	4	
**CHEMBL1935443**	C20H23N3O	5.68	0	4	
**CHEMBL4450665**	C23H28N2O2	5.92	0	4	
**CHEMBL3696120**	C22H26ClN3	5.96	0	4	
**CHEMBL460893**	C23H25N3O	6.13	0	4	
**CHEMBL1346**	C28H30N2O2	7.10	0	4	
**CHEMBL2046892**	C22H22F2N2	7.40	0	4	
**CHEMBL96153**	C24H31NO3	7.52	0	4	
**CHEMBL1223951**	C21H24N2O2	9.21	0	4	
**CHEMBL1257938**	C22H26N2O2	9.21	0	4	
**CHEMBL1257937**	C22H26N2O2	9.28	0	4	
**CHEMBL1257820**	C23H28N2O2	9.60	0	4	
**CHEMBL1257821**	C22H26N2O2	9.85	0	4	
**CHEMBL1097**	C15H23N3O2	4.00	0	5	5.22
**CHEMBL4640366**	C33H34N2O3	4.02	0	5	
**CHEMBL1091879**	C19H25N3O2	4.12	0	5	
**CHEMBL561138**	C23H27N3O2	4.14	0	5	
**CHEMBL1258006**	C22H23N3O2	4.27	0	5	
**CHEMBL3787069**	C26H26N2O3	4.35	0	5	

Table S7 (Cont.d)

**CHEMBL2324239**	**C24H18Cl2NO2^{+}**	**4.51**	**0**	**5**	
**CHEMBL1094041**	C29H29N3O2	4.55	0	5	
**CHEMBL4213196**	C25H34N4O	4.62	0	5	
**CHEMBL914**	C32H39NO4	4.67	0	5	
**CHEMBL217133**	C18H22N4O	4.68	0	5	
**CHEMBL429115**	C20H24N4O	4.71	0	5	
**CHEMBL2441636**	C22H33N3O2	4.72	0	5	
**CHEMBL1080790**	C16H22Cl2N2O	4.78	0	5	
**CHEMBL4096683**	C20H22N4O	4.94	0	5	
**CHEMBL237191**	C25H31N5	4.98	0	5	
**CHEMBL565829**	C15H14N4O	5.01	0	5	
**CHEMBL2177304**	C27H24FN3O	5.07	0	5	
**CHEMBL487063**	C23H27N3O2	5.10	0	5	
**CHEMBL378547**	C21H21F2NO2	5.11	0	5	
**CHEMBL1080969**	C16H22Cl2N2O	5.11	0	5	
**CHEMBL485242**	C25H29N3O2	5.14	0	5	
**CHEMBL478615**	C23H27N3O2	5.17	0	5	
**CHEMBL476579**	C24H29N3O2	5.18	0	5	
**CHEMBL479242**	C23H27N3O2	5.18	0	5	
**CHEMBL3121096**	C20H25N3O2	5.22	0	5	
**CHEMBL498572**	C19H22N2O3	5.23	0	5	
**CHEMBL1819137**	C22H25N3O2	5.23	0	5	
**CHEMBL488249**	C24H27N3O2	5.25	0	5	
**CHEMBL4100591**	C27H34F2N2O	5.29	0	5	
**CHEMBL384400**	C27H36ClN3O	5.48	0	5	
**CHEMBL487064**	C23H27N3O2	5.55	0	5	
**CHEMBL1110**	C17H18N4O	5.64	0	5	
**CHEMBL478616**	C23H27N3O2	5.80	0	5	
**CHEMBL196983**	C21H27NO4	6.10	0	5	

Table S7 (Cont.d)

CHEMBL562833	C22H30ClN3O	6.10	0	5	
**CHEMBL58387**	C31H38N4O	6.24	0	5	
**CHEMBL3775729**	C26H28N4O	6.28	0	5	
**CHEMBL376488**	C32H31BrN2O2	6.43	0	5	
**CHEMBL1671893**	C23H30N2O3	6.60	0	5	
**CHEMBL1087493**	C17H26N4O	6.63	0	5	
**CHEMBL42**	C18H19ClN4	6.72	0	5	
**CHEMBL998**	C22H23ClN2O2	6.77	0	5	
**CHEMBL2324243**	C26H32NO4^{+}	6.80	0	5	
**CHEMBL2324245**	C24H26Cl2NO2^{+}	7.22	0	5	
**CHEMBL572163**	C26H31NO4	7.42	0	5	
**CHEMBL61301**	C19H21FN4	7.56	0	5	
**CHEMBL1642487**	C28H28N2O3	9.09	0	5	
**CHEMBL1642479**	C28H28N2O3	9.17	0	5	
**CHEMBL259732**	C20H27N3O2	9.19	0	5	
**CHEMBL410832**	C23H25N3O2	9.39	0	5	
**CHEMBL4787638**	C21H18ClN3O2	2.08	0	6	**5.88**
**CHEMBL270190**	C25H32N2O4	3.10	0	6	
**CHEMBL519266**	C22H28N2O4	3.11	0	6	
**CHEMBL1688**	C18H19ClN4O	3.85	0	6	
**CHEMBL3741589**	C18H17Cl2NO3	4.11	0	6	
**CHEMBL1000**	C21H25ClN2O3	4.52	0	6	
**CHEMBL4212656**	C24H31N5O	4.64	0	6	
**CHEMBL398612**	C26H35FN4O	4.88	0	6	
**CHEMBL1823042**	C20H28FN3O2	5.00	0	6	
**CHEMBL494406**	C21H24ClN5	5.05	0	6	
**CHEMBL4067770**	C20H21FN4O	5.11	0	6	
**CHEMBL1938433**	C23H24N2O4	5.42	0	6	
**CHEMBL2177904**	C23H16FN5	5.51	0	6	

Table S7 (Cont.d)

**CHEMBL1823047**	C22H30N4O2	5.54	0	6	
**CHEMBL4065208**	C23H22N4O2	5.56	0	6	
**CHEMBL2146805**	C20H21ClN4O	5.60	0	6	
**CHEMBL2177905**	C24H16F2N4	5.80	0	6	
**CHEMBL2147024**	C22H18N6	5.96	0	6	
**CHEMBL563926**	C21H26ClN3O2	6.20	0	6	
**CHEMBL1083463**	C26H26Cl2N4	6.22	0	6	
**CHEMBL4081790**	C25H19N5O	6.30	0	6	
**CHEMBL723**	C24H26N2O4	6.46	0	6	
**CHEMBL4096145**	C22H27N5O	6.52	0	6	
**CHEMBL3774817**	C25H25FN4O	6.59	0	6	
**CHEMBL522456**	CHEMBL522456	7.00	0	6	
**CHEMBL3775282**	C26H28N4O2	7.16	0	6	
**CHEMBL1108**	C22H22FN3O2	7.49	0	6	
**CHEMBL329067**	C32H33N5O	7.62	0	6	
**CHEMBL12780**	C24H27FN4O	8.16	0	6	
**CHEMBL2146810**	C23H27ClN4O	8.40	0	6	
**CHEMBL1423**	C28H29F2N3O	8.52	0	6	
**CHEMBL296419**	C28H31FN4O	9.05	0	6	
**CHEMBL1258503**	C22H25N3O3	9.14	0	6	
**CHEMBL1258280**	C22H24N2O4	9.21	0	6	
**CHEMBL374731**	C10H14N2O5	2.30	0	7	**5.21**
**CHEMBL8**	C17H18FN3O3	3.02	0	7	
**CHEMBL4855895**	C21H24FN3O3	3.53	0	7	
**CHEMBL22**	C14H18N4O3	3.62	0	7	
**CHEMBL3582294**	C25H18F2N4O	4.03	0	7	
**CHEMBL4224807**	C14H16N6O	4.03	0	7	
**CHEMBL388978**	C28H26N4O3	4.04	0	7	
**CHEMBL4847738**	C20H23N3O4	4.05	0	7	

Table S7 (Cont.d)

**CHEMBL4781551**	**C24H21ClN4O2**	**4.08**	**0**	**7**	
**CHEMBL3329814**	C21H28N2O5	4.09	0	7	
**CHEMBL4127458**	C22H22ClN5O	4.22	0	7	
**CHEMBL474484**	C13H21N7	4.28	0	7	
**CHEMBL583**	C19H22FN3O3	4.30	0	7	
**CHEMBL218836**	C18H24F3N3O	4.40	0	7	
**CHEMBL2158823**	C24H28Cl2N2O3	4.50	0	7	
**CHEMBL1957012**	C26H29N5O2	4.60	0	7	
**CHEMBL512975**	C21H23ClFN5	4.64	0	7	
**CHEMBL2333636**	C24H28F4N2O	4.75	0	7	
**CHEMBL2180842**	C17H22FN5O	4.80	0	7	
**CHEMBL2314058**	C25H34F3N3O	4.83	0	7	
**CHEMBL523374**	C22H23N5O2	4.89	0	7	
**CHEMBL3115194**	C26H32BrN3O3	4.90	0	7	
**CHEMBL2333616**	C20H22ClF3N2O	4.92	0	7	
**CHEMBL3218884**	C28H33N5O2	4.96	0	7	
**CHEMBL1957792**	C27H30ClN3O3	4.99	0	7	
**CHEMBL1092106**	C20H24ClFN4O	5.04	0	7	
**CHEMBL2333607**	C25H30F4N2O	5.05	0	7	
**CHEMBL2333615**	C23H26F4N2O	5.05	0	7	
**CHEMBL498042**	C24H23N5O2	5.08	0	7	
**CHEMBL3703271**	C22H23N3O4	5.08	0	7	
**CHEMBL3262625**	C23H26N6O	5.09	0	7	
**CHEMBL246815**	C22H26FN3O3	5.13	0	7	
**CHEMBL523821**	C21H15F2N5	5.16	0	7	
**CHEMBL398478**	C25H28FN3O3	5.21	0	7	
**CHEMBL2178522**	C23H19FN6	5.28	0	7	
**CHEMBL2158815**	C24H28Cl2N2O3	5.30	0	7	
**CHEMBL497048**	C27H23N5O2	5.36	0	7	

Table S7 (Cont.d)

CHEMBL2147022	C20H17N7	5.37	0	7	
**CHEMBL2336331**	C24H29N5O2	5.40	0	7	
**CHEMBL1091834**	C25H26ClFN4O	5.42	0	7	
**CHEMBL1813015**	C20H20N6O	5.42	0	7	
**CHEMBL1939739**	C32H35FN4O2	5.44	0	7	
**CHEMBL250533**	C25H28ClN3O3	5.48	0	7	
**CHEMBL4102707**	C20H20ClF4NO	5.54	0	7	
**CHEMBL2147021**	C20H15FN6	5.57	0	7	
**CHEMBL246634**	C21H23FN2O4	5.58	0	7	
**CHEMBL1098847**	C24H21Cl3N2O2	5.58	0	7	
**CHEMBL2151218**	C25H22FN5O	5.70	0	7	
**CHEMBL3764774**	C28H28N4O3	5.70	0	7	
**CHEMBL3086038**	C30H33BrClN3O2	5.82	0	7	
**CHEMBL3806193**	C19H23Cl2N3O2	5.84	0	7	
**CHEMBL2146812**	C19H20ClN5O	5.90	0	7	
**CHEMBL17423**	C22H25N3O4	5.96	0	7	
**CHEMBL3775387**	C25H27N5O2	5.99	0	7	
**CHEMBL3806153**	C18H21ClFN3O2	6.01	0	7	
**CHEMBL2387265**	C31H29FN2O4	6.09	0	7	
**CHEMBL3775050**	C25H24F2N4O	6.20	0	7	
**CHEMBL45816**	C29H38FN3O3	6.24	0	7	
**CHEMBL501480**	C29H25N5O2	6.50	0	7	
**CHEMBL1084400**	C20H21F3N4	6.80	0	7	
**CHEMBL526466**	C25H23N5O2	7.10	0	7	
**CHEMBL2146854**	C23H26N4O3	7.33	0	7	
**CHEMBL1107**	C26H30Cl2F3NO	7.40	0	7	
**CHEMBL384487**	C24H23FN2O4	7.69	0	7	
**CHEMBL4279819**	C16H18N6O	8.07	0	7	
**CHEMBL217593**	C23H23FN2O4	8.27	0	7	

Table S7 (Cont.d)

**CHEMBL12713**	**C24H26ClFN4O**	**8.57**	**0**	**7**	
**CHEMBL33**	C18H20FN3O4	3.04	0	8	**5.10**
**CHEMBL399726**	C20H24N6O2	3.78	0	8	
**CHEMBL31**	C19H22FN3O4	3.89	0	8	
**CHEMBL3804950**	C20H21F2N5O	4.01	0	8	
**CHEMBL4858551**	C19H19FN6O	4.01	0	8	
**CHEMBL520463**	C28H24N4O4	4.05	0	8	
**CHEMBL485123**	C20H23F3N4O	4.07	0	8	
**CHEMBL32**	C21H24FN3O4	4.10	0	8	
**CHEMBL2333619**	C22H27F4N3O	4.10	0	8	
**CHEMBL2180070**	C19H23N7O	4.27	0	8	
**CHEMBL193**	C17H18N2O6	4.30	0	8	
**CHEMBL2382343**	C29H32F2N2O4	4.32	0	8	
**CHEMBL2314064**	C24H32F3N3O2	4.41	0	8	
**CHEMBL540634**	C26H31Cl2N3O3	4.50	0	8	
**CHEMBL3577935**	C23H34F3N3O2	4.50	0	8	
**CHEMBL3787345**	C25H28N4O4	4.51	0	8	
**CHEMBL2164565**	C19H18F3N3O2	4.63	0	8	
**CHEMBL3216126**	C25H35Cl2N5O	4.65	0	8	
**CHEMBL602875**	C26H34N4O4	4.72	0	8	
**CHEMBL2178221**	C28H29ClN4O3	4.82	0	8	
**CHEMBL3612814**	C24H28N4O4	4.85	0	8	
**CHEMBL3593771**	C19H19N7O	4.87	0	8	
**CHEMBL1093696**	C24H25ClFN5O	4.92	0	8	
**CHEMBL515001**	C21H25FN6O	4.96	0	8	
**CHEMBL1084617**	C24H28N4O4	4.96	0	8	
**CHEMBL3964723**	C23H27Cl2N3O3	5.00	0	8	
**CHEMBL465417**	C25H23N5O3	5.04	0	8	
**CHEMBL3786346**	C25H26N4O4	5.05	0	8	

Table S7 (Cont.d)

**CHEMBL2325997**	**C20H23ClN6O**	**5.14**	**0**	**8**	
**CHEMBL506163**	C22H19ClFN5O	5.20	0	8	
**CHEMBL262341**	C33H29N7O	5.25	0	8	
**CHEMBL2158771**	C27H31Cl2N3O3	5.30	0	8	
**CHEMBL2181489**	C23H20F3N3O2	5.52	0	8	
**CHEMBL3218650**	C27H30ClN5O2	5.60	0	8	
**CHEMBL3703237**	C19H17ClN4O3	5.66	0	8	
**CHEMBL399525**	C19H23N7O	5.75	0	8	
**CHEMBL2178521**	C24H21FN6O	5.75	0	8	
**CHEMBL3415593**	C19H26N6O2	5.77	0	8	
**CHEMBL2387229**	C26H26ClFN2O4	5.80	0	8	
**CHEMBL478462**	C25H38N6O2	5.84	0	8	
**CHEMBL1956991**	C23H26ClFN4O2	5.89	0	8	
**CHEMBL2441431**	C22H23N3O5	5.89	0	8	
**CHEMBL1621**	C23H27FN4O3	6.00	0	8	
**CHEMBL3318989**	C23H24ClN5O2	6.00	0	8	
**CHEMBL515025**	C24H36N6O2	6.15	0	8	
**CHEMBL478011**	C25H38N6O2	6.38	0	8	
**CHEMBL94454**	C24H25FN6O	6.46	0	8	
**CHEMBL453894**	C25H26F3N3O2	6.46	0	8	
**CHEMBL233047**	C20H32Cl2N6	6.52	0	8	
**CHEMBL2146813**	C38H48ClN5O2	6.80	0	8	
**CHEMBL568571**	C27H26F3N3O2	6.85	0	8	
**CHEMBL128007**	C28H28Cl2N4O2	7.00	0	8	
**CHEMBL525413**	C29H24ClN5O2	7.09	0	8	
**CHEMBL2146853**	C20H23N5O3	7.60	0	8	
**CHEMBL214021**	C23H21FN2O5	7.82	0	8	
**CHEMBL217789**	C23H21ClN2O5	8.44	0	8	
**CHEMBL2324519**	C20H21FN6O2	3.67	0	9	**5.15**

Table S7 (Cont.d)

**CHEMBL3799831**	**C25H26FN3O5**	**4.03**	**0**	**9**	
**CHEMBL3290351**	C24H27FN6O2	4.05	0	9	
**CHEMBL216323**	C23H19FN2O6	4.07	0	9	
**CHEMBL4092041**	C29H31F3N2O4	4.07	0	9	
**CHEMBL3290344**	C24H26N6O3	4.13	0	9	
**CHEMBL248296**	C27H31F2N3O4	4.19	0	9	
**CHEMBL2165068**	C25H26FN5O3	4.24	0	9	
**CHEMBL484816**	C22H21N7O2	4.30	0	9	
**CHEMBL4094682**	C22H27ClN6O2	4.34	0	9	
**CHEMBL3218891**	C27H32N6O3	4.37	0	9	
**CHEMBL2325729**	C26H34ClN7O	4.53	0	9	
**CHEMBL3650850**	C21H23N5O4	4.68	0	9	
**CHEMBL3262358**	C21H22ClN7O	4.70	0	9	
**CHEMBL3612928**	C24H28N4O5	4.70	0	9	
**CHEMBL3786311**	C26H30N4O5	4.80	0	9	
**CHEMBL596700**	C20H23F3N4O2	4.88	0	9	
**CHEMBL2441417**	C24H26N6O3	4.89	0	9	
**CHEMBL255968**	C25H24ClN5O3	5.00	0	9	
**CHEMBL1095476**	C28H25Cl3N4O2	5.06	0	9	
**CHEMBL3416021**	C20H24N8O	5.07	0	9	
**CHEMBL589833**	C24H25Cl2FN4O2	5.10	0	9	
**CHEMBL3612926**	C25H30N4O5	5.11	0	9	
**CHEMBL2164393**	C26H24F3N3O3	5.14	0	9	
**CHEMBL2170611**	C17H21F3N4O2	5.16	0	9	
**CHEMBL429458**	C25H30FN3O5	5.20	0	9	
**CHEMBL4075071**	C19H16F3N5O	5.25	0	9	
**CHEMBL4170966**	C24H21Cl2N3O4	5.33	0	9	
**CHEMBL2164389**	C24H20F3N3O3	5.34	0	9	
**CHEMBL255645**	C28H31Cl2N5O2	5.36	0	9	

Table S7 (Cont.d)

**CHEMBL1927161**	**C24H29N5O4**	**5.40**	**0**	**9**	
**CHEMBL489324**	C28H42ClF2N5O	5.68	0	9	
**CHEMBL3237444**	C23H25ClN8	5.70	0	9	
**CHEMBL4213486**	C19H15F2N5O2	5.70	0	9	
**CHEMBL429761**	C23H31N7O2	5.73	0	9	
**CHEMBL3318999**	C28H25N7O2	5.85	0	9	
**CHEMBL2177305**	C28H23ClFN5O2	5.89	0	9	
**CHEMBL446966**	C34H31N7O2	5.91	0	9	
**CHEMBL550410**	C25H26F4N4O	5.92	0	9	
**CHEMBL557306**	C21H24ClF3N2O3	5.99	0	9	
**CHEMBL514042**	C25H38N6O3	6.05	0	9	
**CHEMBL549635**	C29H33F4N3O2	6.20	0	9	
**CHEMBL498411**	C22H19Cl3N6	6.46	0	9	
**CHEMBL217442**	C24H23FN2O6	7.80	0	9	
**CHEMBL1729**	C23H29ClFN3O4	8.19	0	9	
**CHEMBL387178**	C23H20F2N2O5	8.21	0	9	
**CHEMBL3422973**	C27H28N6O3	8.68	0	9	
**CHEMBL3422970**	C27H28N6O3	9.06	0	9	
**CHEMBL2424928**	C24H26FN5O4	3.51	0	10	**5.24**
**CHEMBL1916543**	C23H28N6O4	3.69	0	10	
**CHEMBL605785**	C27H27FN4O5	4.02	0	10	
**CHEMBL3221500**	C22H21ClN6O3	4.20	0	10	
**CHEMBL2151322**	C19H20FN7O2	4.28	0	10	
**CHEMBL3794265**	C23H25FN6O3	4.38	0	10	
**CHEMBL247690**	C29H33F2N3O5	4.47	0	10	
**CHEMBL3221488**	C23H23ClN6O3	4.50	0	10	
**CHEMBL3425929**	C28H33FN6O3	4.51	0	10	
**CHEMBL2041188**	C32H30N6O4	4.64	0	10	
**CHEMBL2324520**	C20H20F2N6O2	4.68	0	10	

Table S7 (Cont.d)

**CHEMBL2315921**	**C26H28F3N5O2**	**4.77**	**0**	**10**	
**CHEMBL563791**	C28H29F3N6O	4.90	0	10	
**CHEMBL3400819**	C26H26FN5O4	4.91	0	10	
**CHEMBL256060**	C26H26ClN5O4	4.96	0	10	
**CHEMBL595944**	C27H26N8O2	4.98	0	10	
**CHEMBL2164365**	C23H19F3N4O3	5.04	0	10	
**CHEMBL2177736**	C19H23N9O	5.07	0	10	
**CHEMBL3681314**	C26H25F2N7O	5.07	0	10	
**CHEMBL513921**	C23H34N6O4	5.24	0	10	
**CHEMBL562285**	C24H27N3O2	5.27	0	10	
**CHEMBL2164047**	C24H19F4N3O3	5.28	0	10	
**CHEMBL560063**	C24H23Cl2F3N4O	5.46	0	10	
**CHEMBL245642**	C25H25F2N3O5	5.50	0	10	
**CHEMBL551281**	C26H34F3N5O2	5.52	0	10	
**CHEMBL1236904**	C26H31BrN8O	5.52	0	10	
**CHEMBL3604800**	C28H32FN5O4	5.52	0	10	
**CHEMBL452823**	C37H38N10	5.67	0	10	
**CHEMBL553196**	C32H32F4N4O2	5.76	0	10	
**CHEMBL3323074**	C32H28FN7O2	5.85	0	10	
**CHEMBL3323073**	C32H28FN7O2	6.20	0	10	
**CHEMBL537847**	C23H29Cl2N5O3	6.25	0	10	
**CHEMBL3605145**	C29H32FN5O4	6.30	0	10	
**CHEMBL3605140**	C27H30FN5O4	6.40	0	10	
**CHEMBL375330**	C34H38ClF3N4O2	6.77	0	10	
**CHEMBL4081080**	C25H17F6N3O	7.17	0	10	
**CHEMBL428594**	C29H33F7N2O	7.80	0	10	
**CHEMBL217707**	C23H19F3N2O5	8.25	0	10	
**CHEMBL213715**	C24H22F2N2O6	8.44	0	10	
**CHEMBL3964789**	C21H22F4N6O	3.17	0	11	**5.00**

Table S7 (Cont.d)

CHEMBL3400817	C26H26FN5O5	3.76	0	11	
**CHEMBL2165057**	C25H30N6O5	4.06	0	11	
**CHEMBL1916544**	C22H27N7O4	4.09	0	11	
**CHEMBL3221490**	C23H22ClN7O3	4.40	0	11	
**CHEMBL3703025**	C22H25F3N6O2	4.50	0	11	
**CHEMBL1091218**	C29H25F3N2O6	4.58	0	11	
**CHEMBL2069410**	C23H19F4N3O4	4.60	0	11	
**CHEMBL4209441**	C26H23FN8O2	4.62	0	11	
**CHEMBL4211893**	C30H29FN8O2	4.87	0	11	
**CHEMBL3221503**	C23H20ClN7O3	4.90	0	11	
**CHEMBL2164375**	C25H26F3N3O5	4.96	0	11	
**CHEMBL2204270**	C24H26F4N4O3	5.00	0	11	
**CHEMBL3318984**	C23H23F3N6O2	5.00	0	11	
**CHEMBL508098**	C36H37N11	5.08	0	11	
**CHEMBL3353404**	C26H29ClN8O2	5.17	0	11	
**CHEMBL3746204**	C22H17F4N5O2	5.24	0	11	
**CHEMBL2331648**	C20H22F3N5O3	5.34	0	11	
**CHEMBL2147223**	C22H20ClF3N4O3	5.40	0	11	
**CHEMBL3422758**	C27H29F2N5O4	5.76	0	11	
**CHEMBL1097456**	C26H22Cl3F3N2O3	5.84	0	11	
**CHEMBL256154**	C27H32F6N4O	5.85	0	11	
**CHEMBL3605123**	C30H36FN5O5	6.16	0	11	
**CHEMBL3422952**	C26H27F2N5O4	9.14	0	11	
**CHEMBL3422978**	C26H26F3N5O3	9.41	0	11	
**CHEMBL254316**	C20H21FN6O5	2.50	0	12	**4.80**
**CHEMBL3425799**	C26H25F2N5O5	3.48	0	12	
**CHEMBL451887**	C40H57N5O7	4.04	0	12	
**CHEMBL3342693**	C23H22F5N5O2	4.08	0	12	
**CHEMBL3425807**	C25H25FN6O5	4.52	0	12	

Table S7 (Cont.d)

CHEMBL2206791	C23H21F3N6O3	4.58	0	12	
CHEMBL2147316	C22H19F5N4O3	4.60	0	12	
CHEMBL2336323	C26H30F2N6O4	4.62	0	12	
CHEMBL2147303	C22H19F5N4O3	4.70	0	12	
CHEMBL402624	C30H34F6N2O4	4.71	0	12	
CHEMBL3221487	C20H19ClN8O3	4.80	0	12	
CHEMBL2064657	C28H23ClF2N6O3	4.91	0	12	
CHEMBL3605131	C29H32FN5O6	4.91	0	12	
CHEMBL3764895	C29H31Cl2F3N4O3	5.13	0	12	
CHEMBL3288030	C44H53FN6O5	5.20	0	12	
CHEMBL3764357	C29H31Cl2F3N4O3	5.20	0	12	
CHEMBL2441412	C24H23F3N6O3	5.29	0	12	
CHEMBL2207664	C22H24Cl2F3N3O4	5.30	0	12	
CHEMBL550535	C27H33F6N5O	5.41	0	12	
CHEMBL3765012	C30H31Cl2F3N4O3	5.51	0	12	
CHEMBL3605006	C31H34FN5O6	5.80	0	12	
CHEMBL195378	C23H24F4N6O3	4.23	0	13	**5.29**
CHEMBL3605022	C29H32FN7O5	4.54	0	13	
CHEMBL370072	C24H24F4N4O5	4.55	0	13	
CHEMBL3218816	C19H17F3N8O2	4.75	0	13	
CHEMBL1099069	C19H24F3N9O	5.29	0	13	
CHEMBL4079820	C26H23F4N7O2	5.62	0	13	
CHEMBL401608	C27H31ClF6N4O2	6.73	0	13	
CHEMBL4077588	C29H29F4N7O2	7.00	0	13	
CHEMBL3422957	C27H27F4N5O4	8.77	0	13	
CHEMBL4209354	C15H20N2O2S	4.07	1	5	**5.90**
CHEMBL1125	C16H18N4S	4.90	1	5	
CHEMBL2326478	C18H18FN3S	5.17	1	5	
CHEMBL1956112	C21H28N4S	5.33	1	5	

Table S7 (Cont.d)

**CHEMBL1224697**	**C22H28N2O2S**	**5.90**	**1**	**5**	
**CHEMBL715**	C17H20N4S	6.74	1	5	
**CHEMBL195180**	C24H27ClN2OS	7.52	1	5	
**CHEMBL1257577**	C19H22N2O2S	9.37	1	5	
**CHEMBL1257578**	C20H24N2O2S	9.59	1	5	
**CHEMBL4100776**	C20H22N4OS	5.23	1	6	**5.85**
**CHEMBL225036**	C24H26N4OS	5.29	1	6	
**CHEMBL4089699**	C19H19N3O2S	5.42	1	6	
**CHEMBL4104102**	C20H22N4OS	5.61	1	6	
**CHEMBL3219616**	C24H30N2O3S	5.80	1	6	
**CHEMBL1224699**	C21H26N2O3S	5.90	1	6	
**CHEMBL561279**	C25H34N2O3S	5.93	1	6	
**CHEMBL556100**	C22H25NO4S	6.33	1	6	
**CHEMBL4173253**	C26H34N2O3S	7.10	1	6	
**CHEMBL533**	C20H36N2O3S	8.00	1	6	
**CHEMBL578834**	C21H25N5OS	3.72	1	7	**5.31**
**CHEMBL2069925**	C21H21N5OS	4.52	1	7	
**CHEMBL575241**	C21H26N6S	4.71	1	7	
**CHEMBL3973288**	C20H22FN3O2S	4.84	1	7	
**CHEMBL560386**	C23H29NO5S	5.31	1	7	
**CHEMBL243901**	C23H31N5OS	5.90	1	7	
**CHEMBL1079578**	C28H31N5OS	6.20	1	7	
**CHEMBL708**	C21H21ClN4OS	6.92	1	7	
**CHEMBL584766**	C23H24FN5S	7.00	1	7	
**CHEMBL256653**	C23H26N6OS	4.30	1	8	**5.40**
**CHEMBL1939742**	C30H37N5O2S	4.75	1	8	
**CHEMBL257901**	C28H30N6OS	4.90	1	8	
**CHEMBL270239**	C30H34N6OS	4.90	1	8	
**CHEMBL2440407**	C23H26N2O5S	5.02	1	8	

Table S7 (Cont.d)

**CHEMBL3287218**	**C29H32N6OS**	**5.16**	**1**	**8**	
**CHEMBL207220**	C24H40N4O3S	5.40	1	8	
**CHEMBL270852**	C32H38N6OS	5.40	1	8	
**CHEMBL272086**	C32H38N6OS	5.60	1	8	
**CHEMBL2207738**	C23H26N4O3S	5.60	1	8	
**CHEMBL1080489**	C22H27N5O2S	5.80	1	8	
**CHEMBL408169**	C28H32N6OS	6.40	1	8	
**CHEMBL244280**	C26H28FN5OS	6.60	1	8	
**CHEMBL244278**	C26H28ClN5OS	7.10	1	8	
**CHEMBL397429**	C28H30N6OS	7.30	1	8	
**CHEMBL3608687**	C16H15ClN6OS	4.18	1	9	**5.23**
**CHEMBL4084170**	C15H18F5N3S	4.69	1	9	
**CHEMBL3422244**	C18H18F3N3O2S	4.71	1	9	
**CHEMBL1083118**	C17H19F2N3O3S	4.89	1	9	
**CHEMBL1091664**	C28H25ClN2O5S	5.00	1	9	
**CHEMBL502288**	C23H22N6O2S	5.11	1	9	
**CHEMBL402015**	C27H27BrN6OS	5.20	1	9	
**CHEMBL4243499**	C19H16F3N5O	5.25	1	9	
**CHEMBL2158625**	C20H19Cl2N5OS	5.44	1	9	
**CHEMBL244083**	C29H36N4O4S	5.60	1	9	
**CHEMBL245119**	C25H31N7OS	5.60	1	9	
**CHEMBL2158629**	C20H21Cl2N5OS	5.85	1	9	
**CHEMBL1081747**	C21H23ClFN5OS	6.40	1	9	
**CHEMBL3983509**	C20H20ClFN4O2S	6.70	1	9	
**CHEMBL3921669**	C23H26N4O5S	2.22	1	10	**5.90**
**CHEMBL2440387**	C25H23ClN4O4S	3.38	1	10	
**CHEMBL4112037**	C16H14F2N6OS	4.16	1	10	
**CHEMBL2403108**	C28H36ClN5O3S	5.90	1	10	
**CHEMBL1081867**	C22H23F3N6S	6.33	1	10	

Table S7 (Cont.d)

**CHEMBL401576**	**C25H26F3N5OS**	**6.90**	**1**	**10**	
**CHEMBL390649**	C28H31F3N6S	7.50	1	10	
**CHEMBL3985847**	C21H26N6O4S	2.40	1	11	**5.46**
**CHEMBL1782574**	C23H35F3N4O3S	2.41	1	11	
**CHEMBL1091605**	C29H36F3N3O4S	4.52	1	11	
**CHEMBL2204260**	C22H25F3N4O3S	4.62	1	11	
**CHEMBL3407784**	C20H19ClN6O3S	4.89	1	11	
**CHEMBL4075908**	C20H21BrFN5O3S	5.46	1	11	
**CHEMBL192**	C22H30N6O4S	5.48	1	11	
**CHEMBL2041175**	C31H27N7O3S	5.82	1	11	
**CHEMBL1082111**	C22H23F4N5OS	5.90	1	11	
**CHEMBL402016**	C28H27F3N6OS	6.00	1	11	
**CHEMBL411293**	C28H28F3N7S	6.60	1	11	

Table S8. A Comparative Analysis of Docking Results for Mismatched Molecule Pair between the Open-like and Open Inactivated States of the hERG Channel.

State of the hERG channel	CHEMBL ID	pIC_{50}	Glide (kcal/mol)	
			SP	XP
**Open-Like**	**CHEMBL70**	3.00	-5.33	-5.14
	**CHEMBL1257821**	9.85	-6.84	-6.12
**Open-inactivated**	**CHEMBL70**	3.00	-6.44	-6.77
	**CHEMBL1257821**	9.85	-8.84	-8.27

Supporting Figures

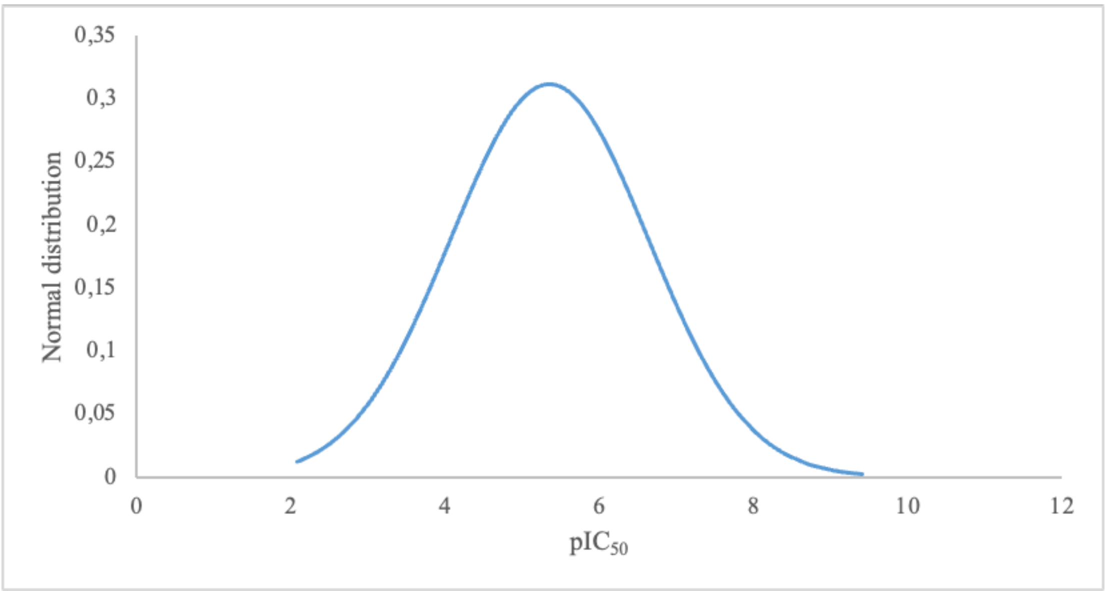

Figure S1. Normal distribution analysis of hERG blockers in a subset 508 compounds

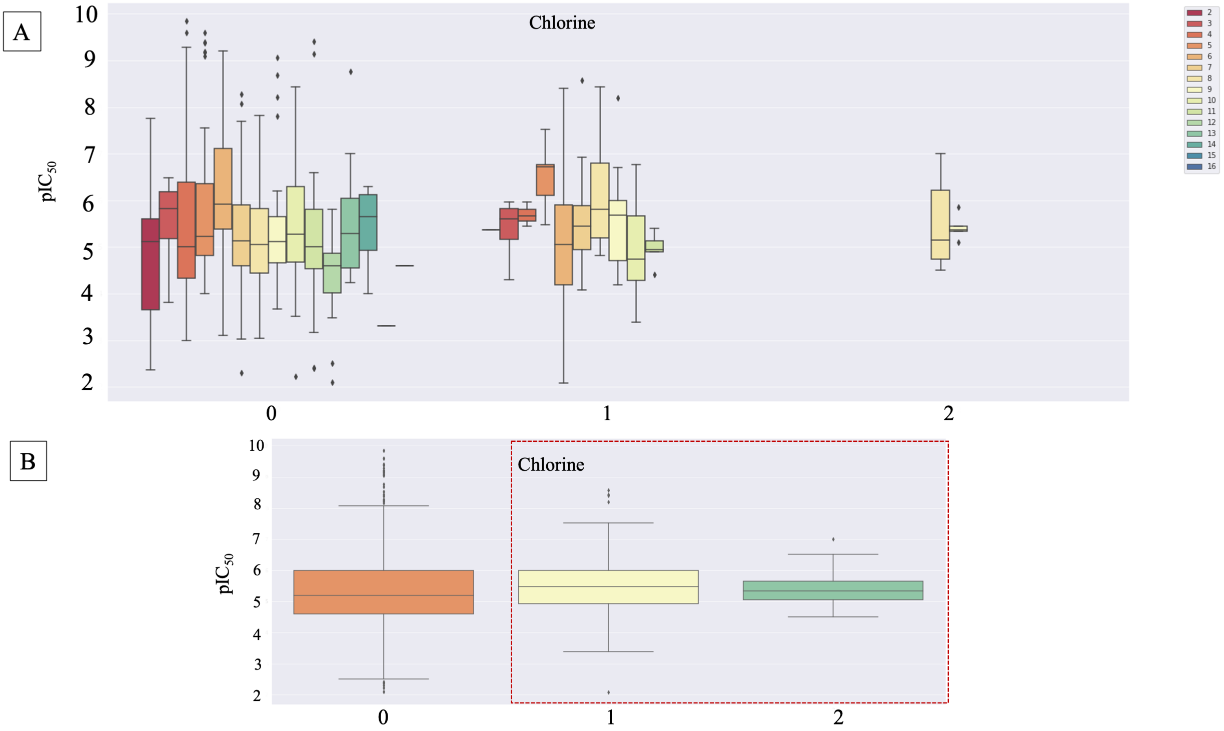

Figure S2. Influence of heteroatoms on the activity of the hERG blockers. A): Represents the influence of the Chlorine on the pIC50, B): Detailed analyzes on the influence of the Chlorine on the pIC50 activity.

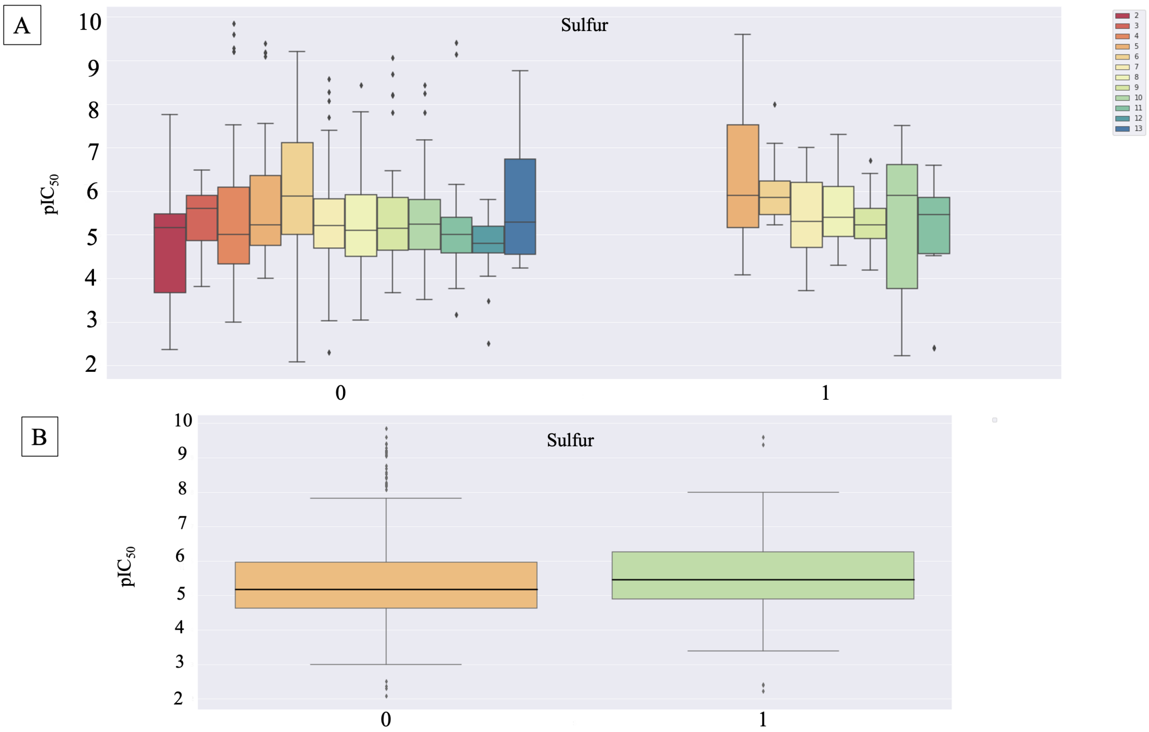

Figure S3. Influence of heteroatoms on the activity of the hERG blockers. A): Represents the influence of the Sulfur on the pIC_{50}, B): Detailed analyzes on the influence of the Sulfur on the pIC_{50} activity.

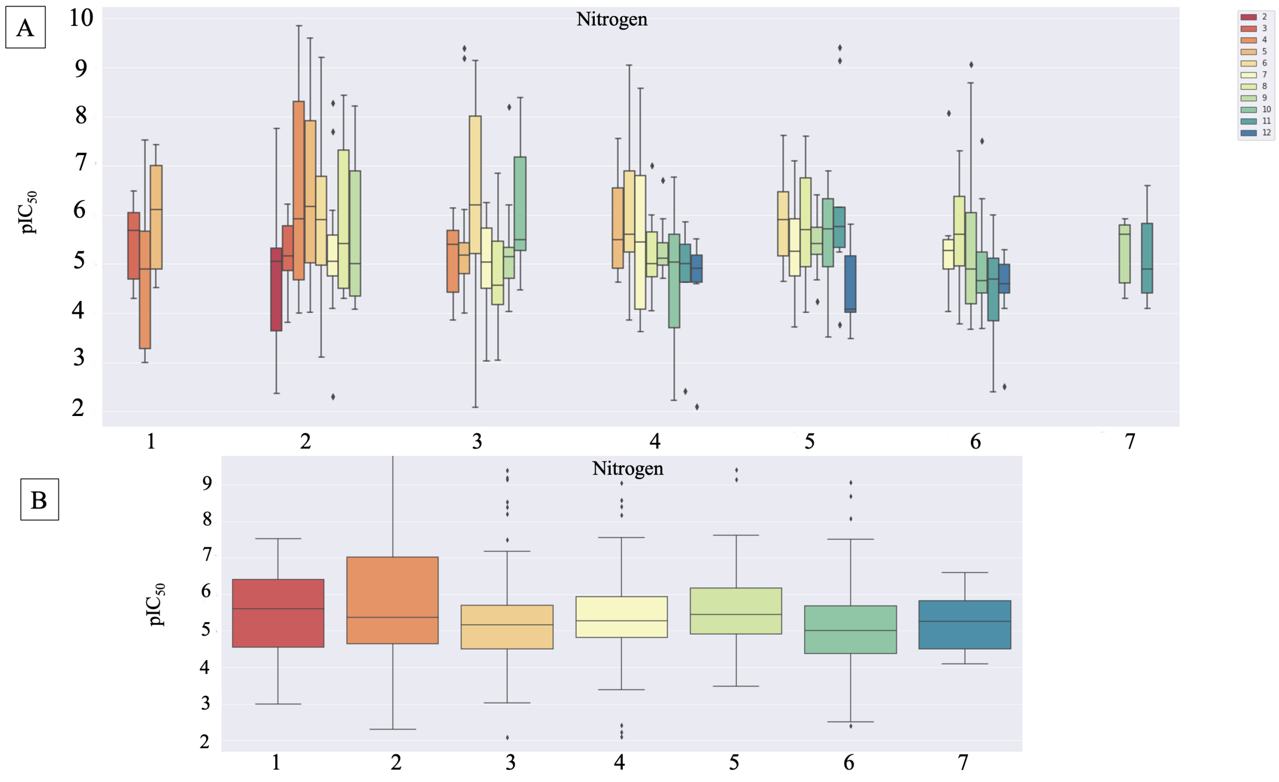

Figure S4. Influence of heteroatoms on the activity of the hERG blockers. A): Represents the influence of the Nitrogen on the pIC_{50}, B): Detailed analyzes on the influence of the Nitrogen on the pIC_{50} activity.

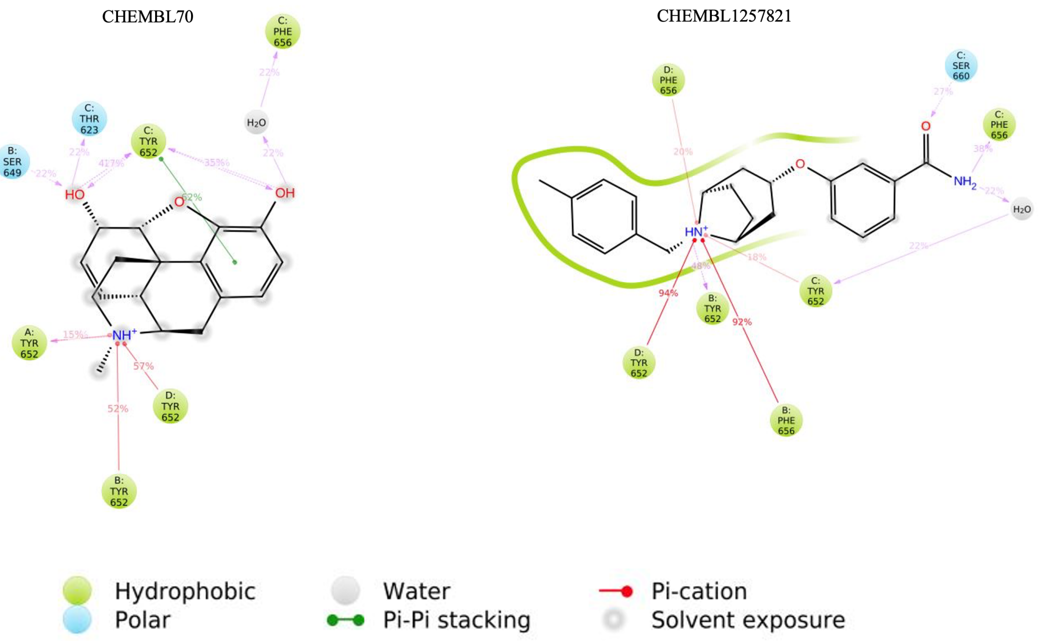

Figure S5. Protein ligand interaction for pair 1.

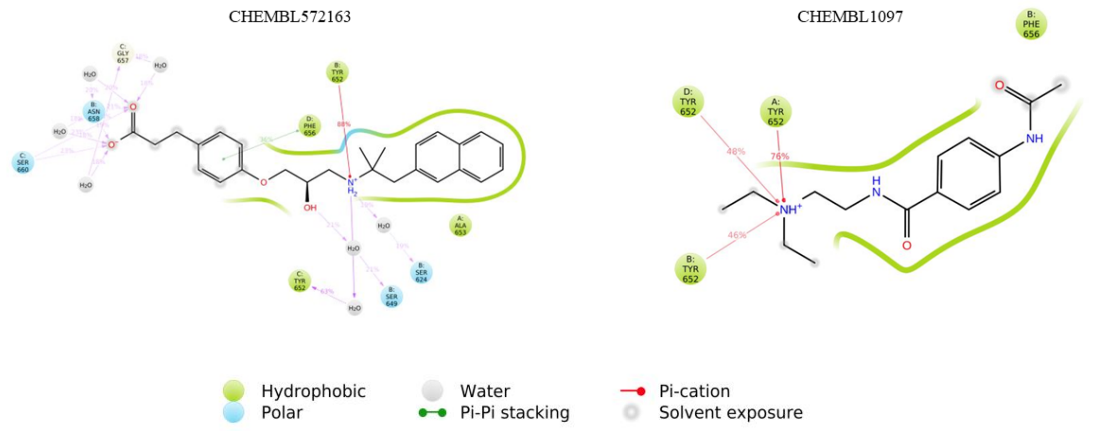

Figure S6. Protein ligand interaction for pair 2.

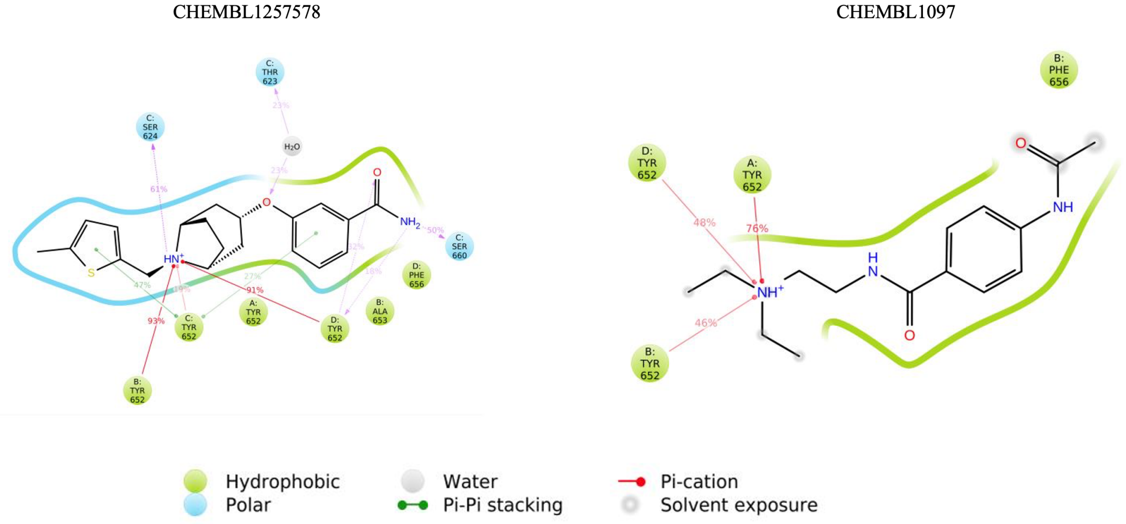

Figure S7. Protein ligand interaction for pair 3.

CHEMBL1782574 CHEMBL3422978

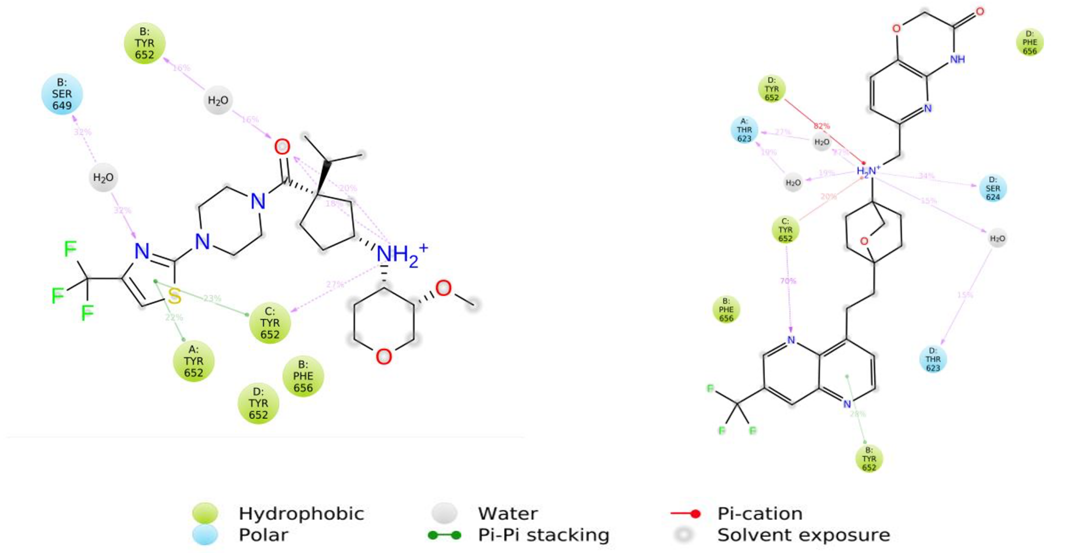

Figure S8. Protein ligand interaction for pair 4.

CHEMBL2424928 CHEMBL3900649

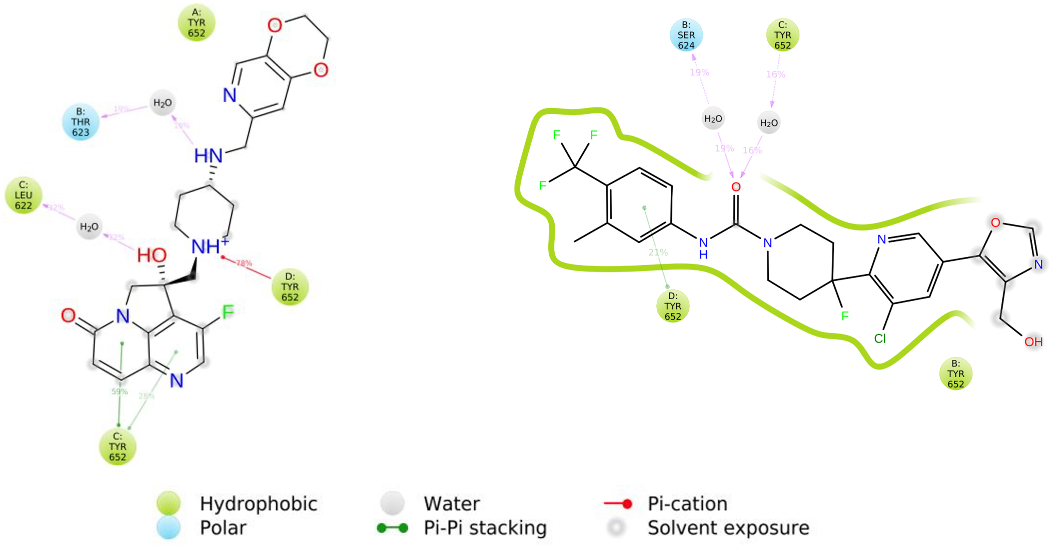

Figure S9. Protein ligand interaction for pair 5.

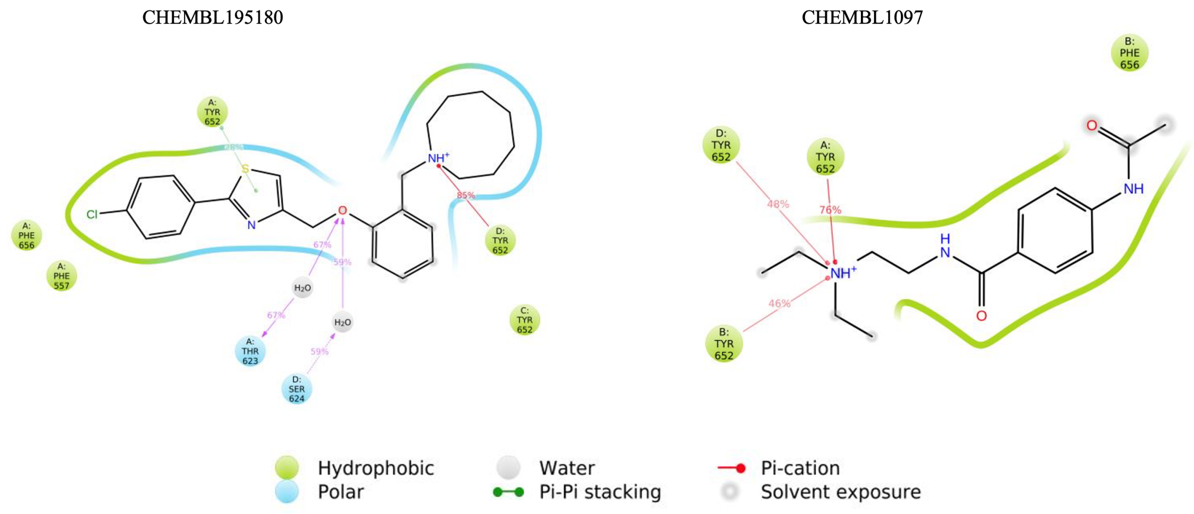

Figure S10. Protein ligand interaction for pair 6.

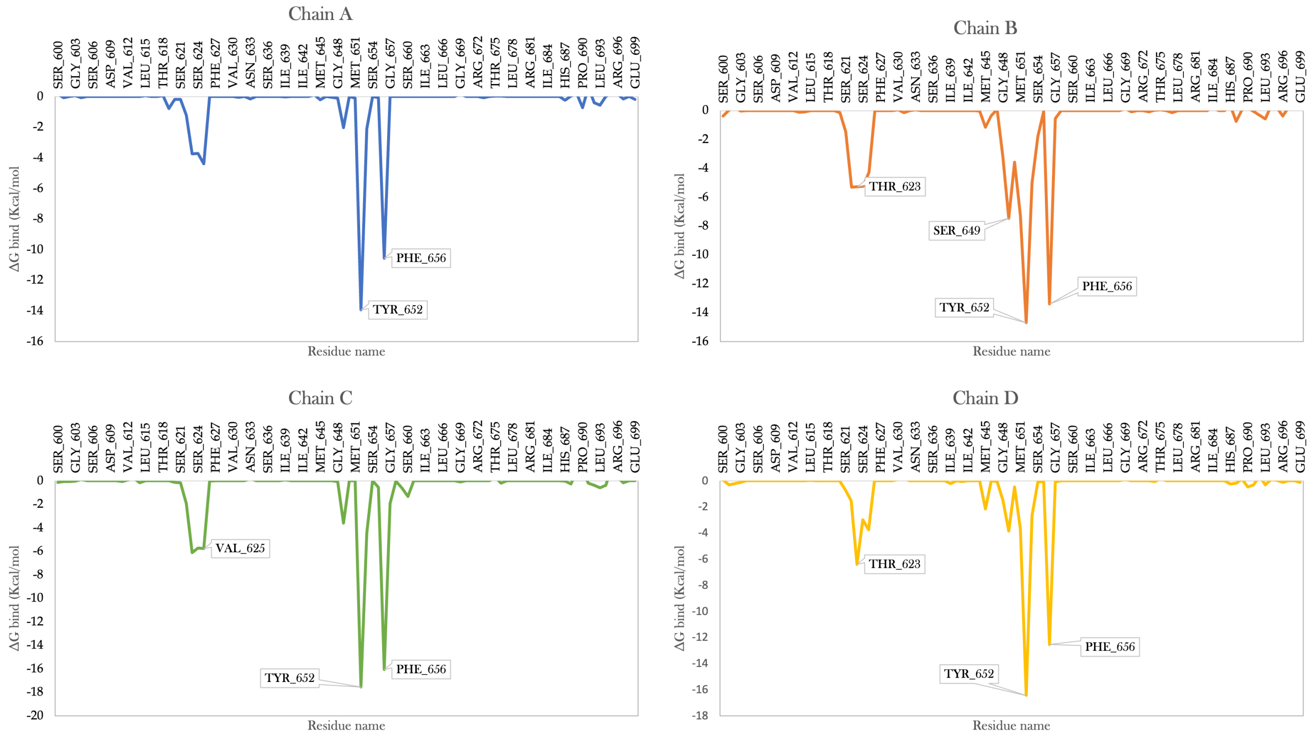

Figure S11. MM/GBSA analysis per-residue for CHEMBL70.

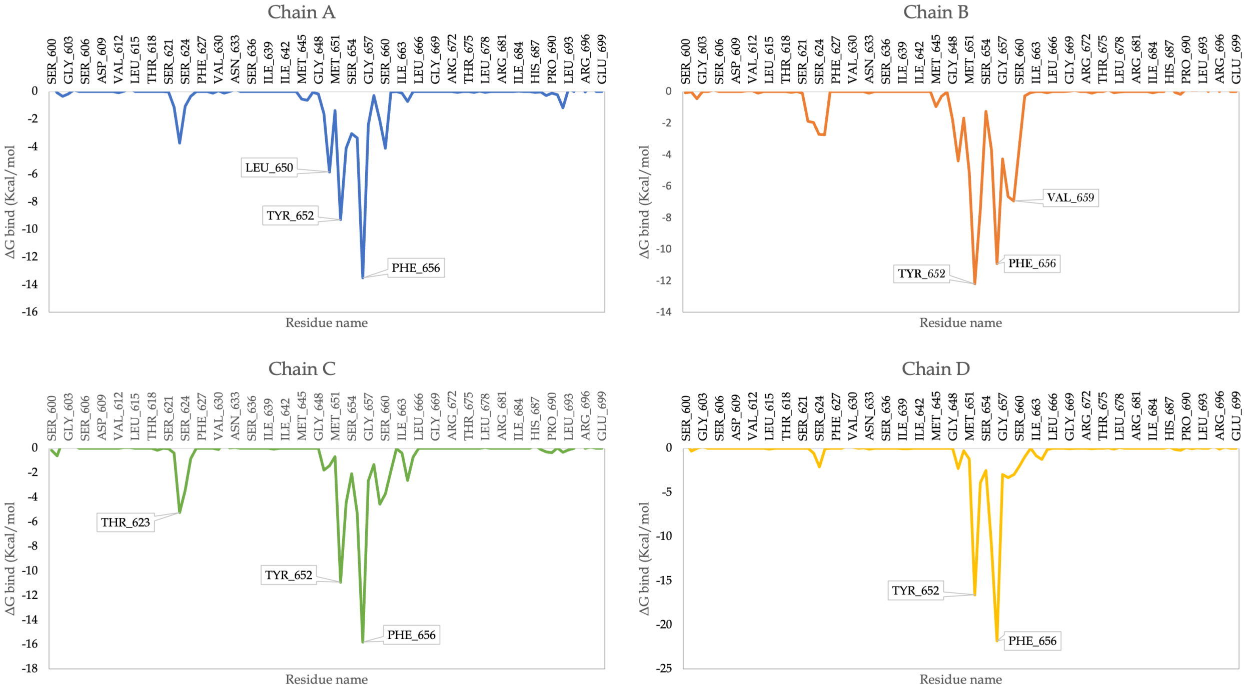

Figure S12. MM/GBSA analysis per-residue for CHEMBL572163.

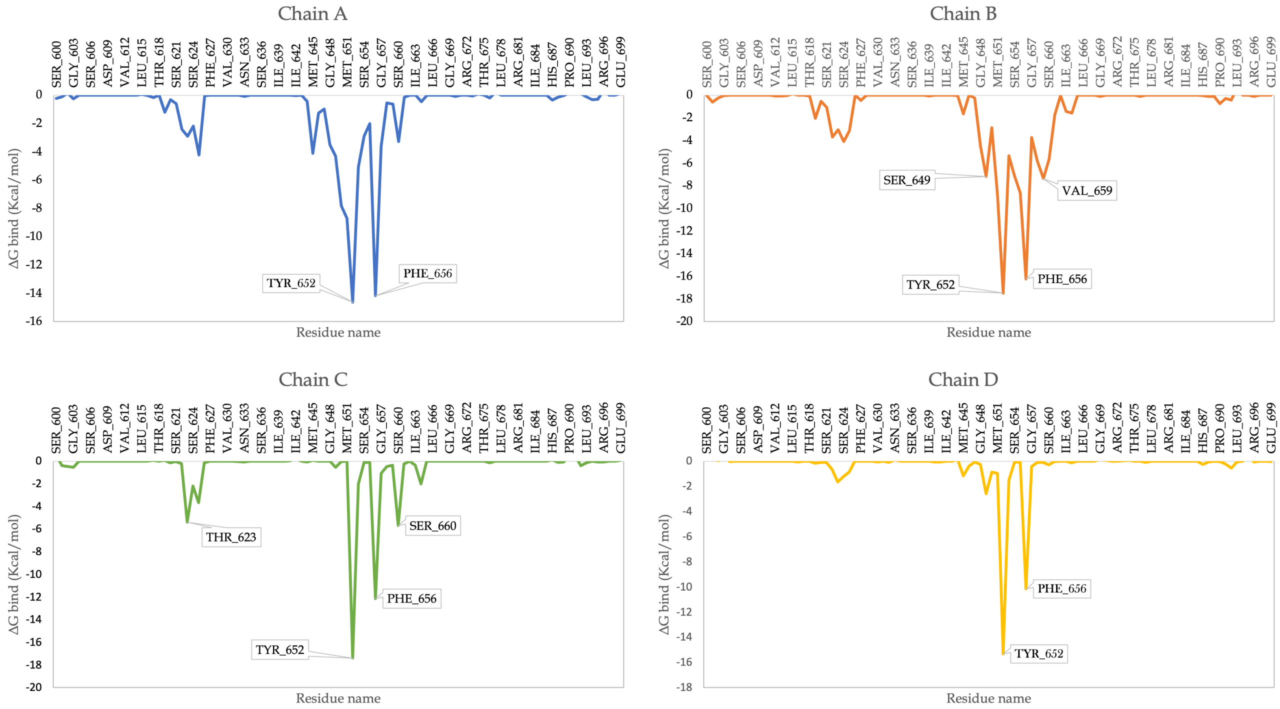

Figure S13. MM/GBSA analysis per-residue for CHEMBL1097.

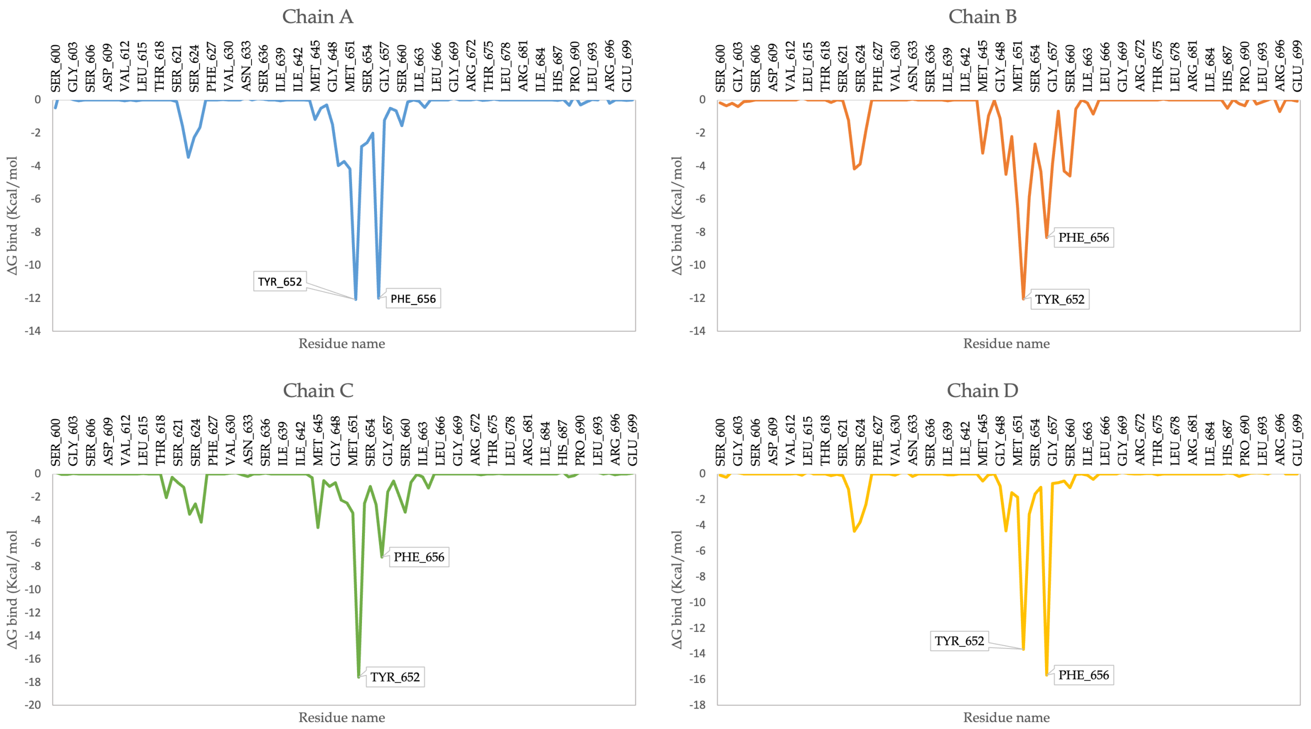

Figure S14. MM/GBSA analysis per-residue for CHEMBL1782574.

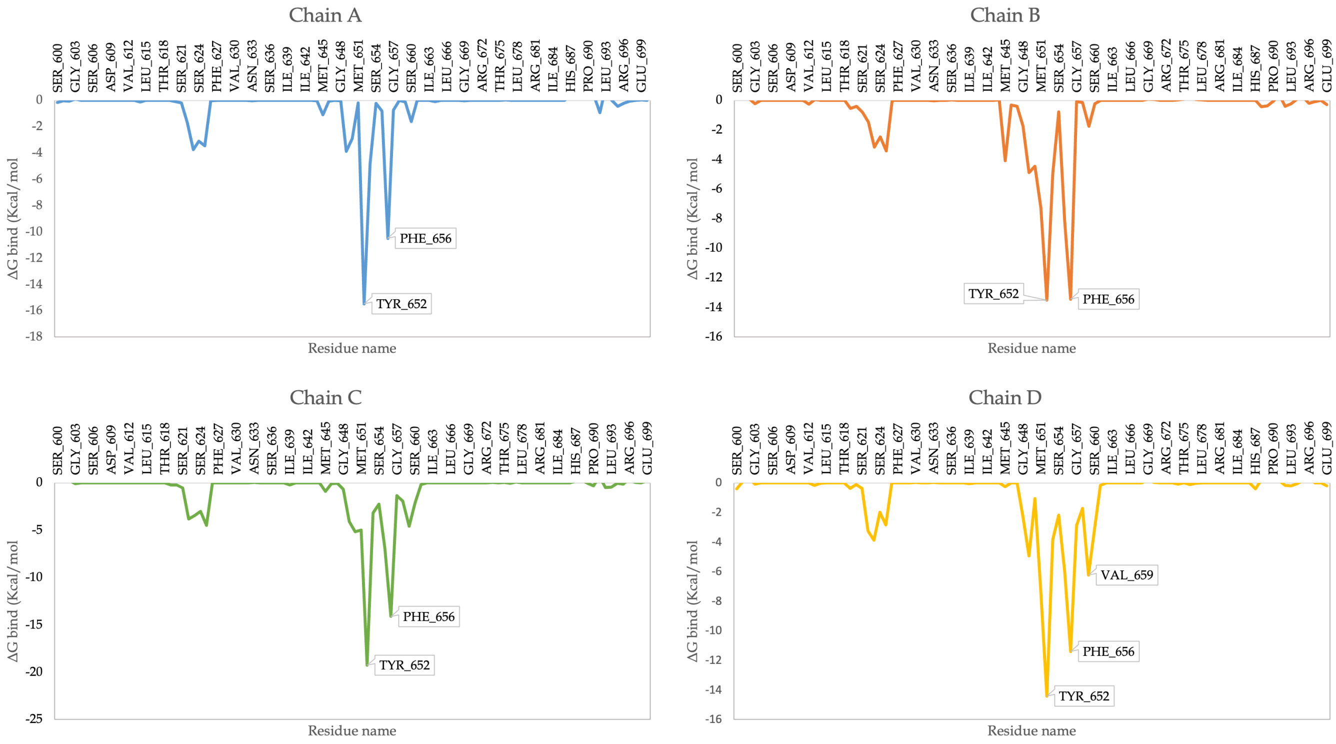

Figure S15. MM/GBSA analysis per-residue for CHEMBL3422978.

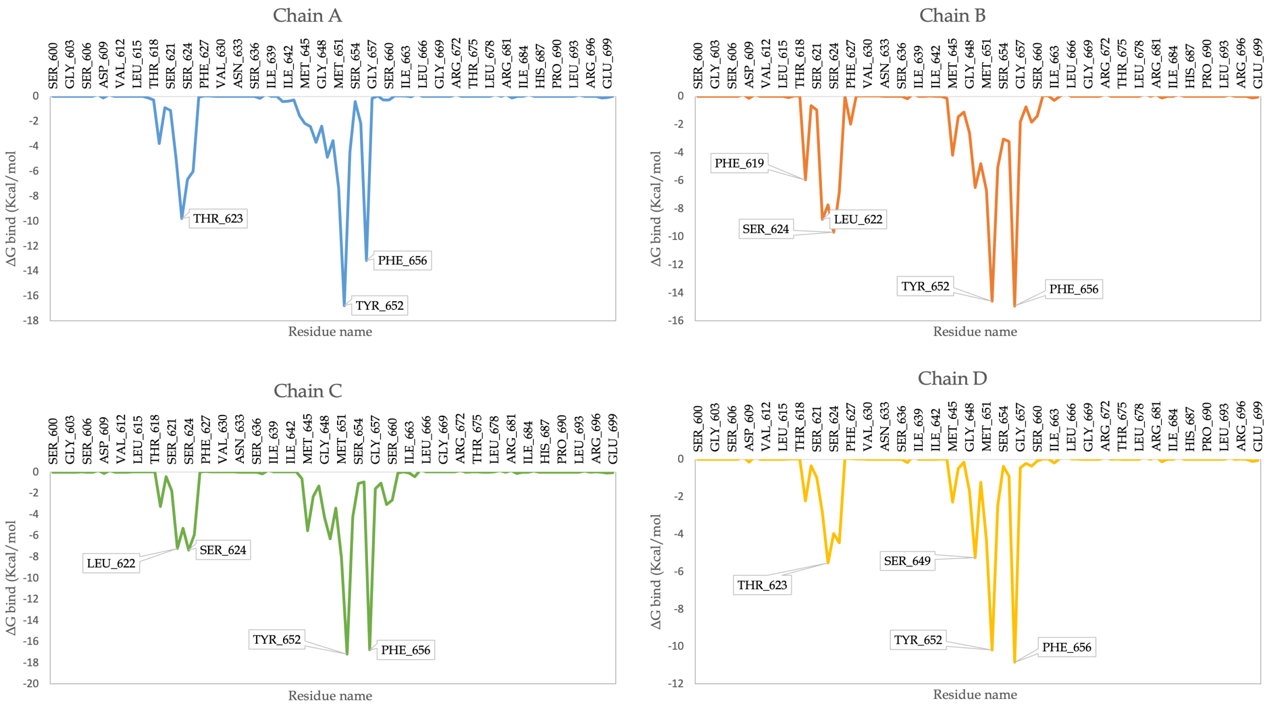

Figure S16. MM/GBSA analysis per-residue for CHEMBL2424928.

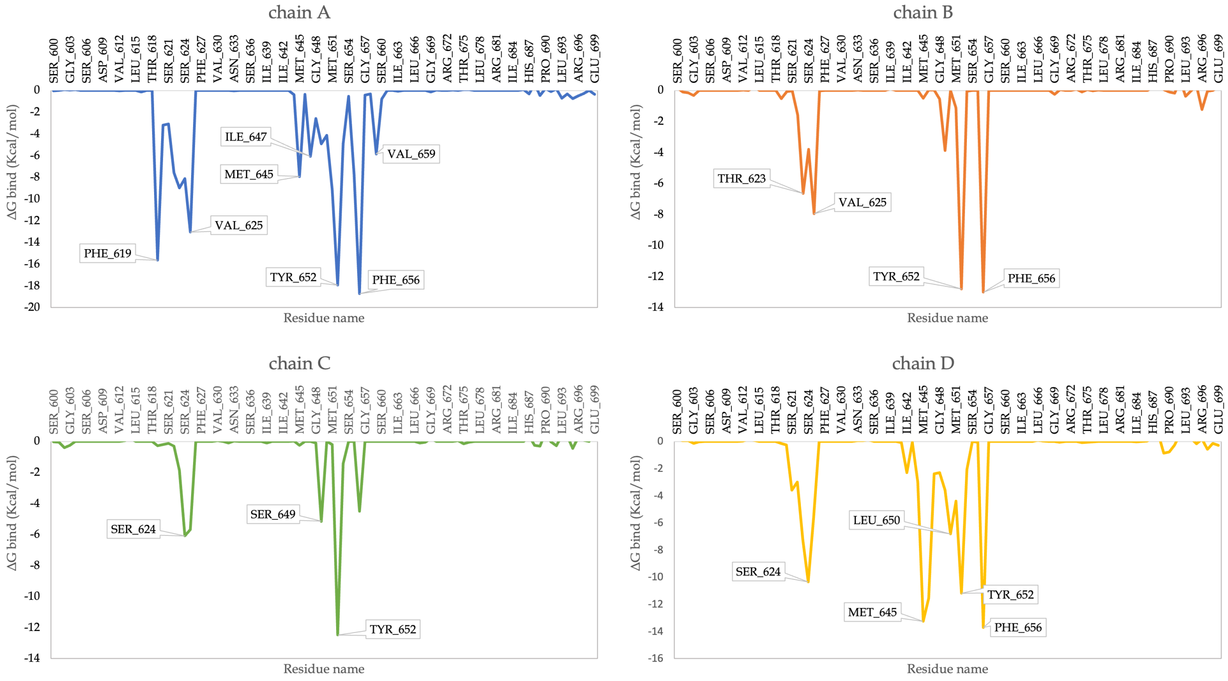

Figure S17. MM/GBSA analysis per-residue for CHEMBL195180.

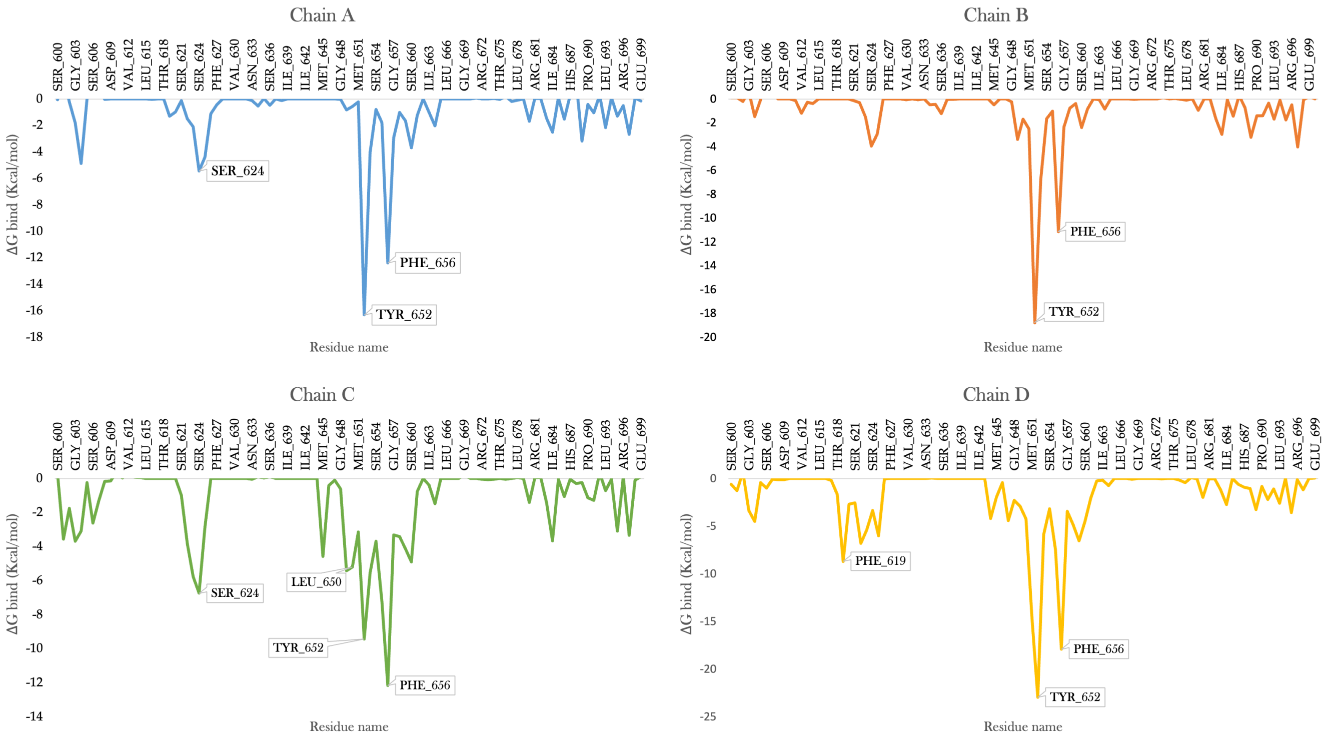

Figure S18. MM/GBSA analysis per-residue for CHEMBL390649.

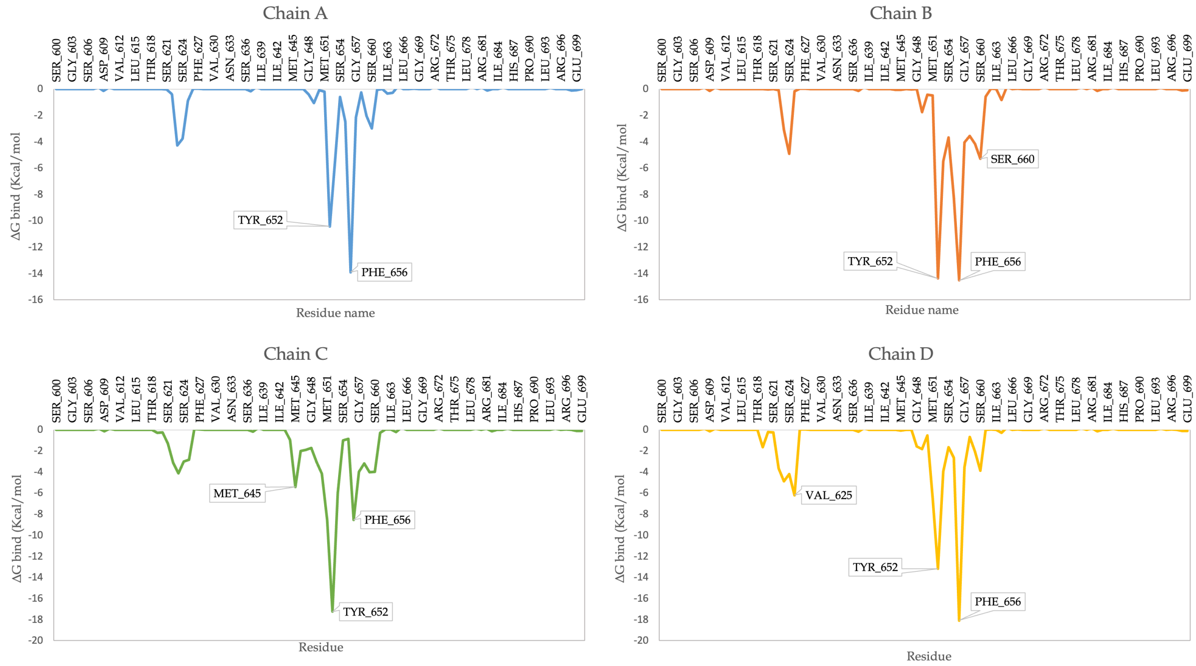

Figure S19. MM/GBSA analysis per-residue for CHEMBL1257821.
